# Where is the UK's pollinator biodiversity? The importance of urban areas for flower-visiting insects

**DOI:** 10.1098/rspb.2014.2849

**Published:** 2015-03-22

**Authors:** Katherine C. R. Baldock, Mark A. Goddard, Damien M. Hicks, William E. Kunin, Nadine Mitschunas, Lynne M. Osgathorpe, Simon G. Potts, Kirsty M. Robertson, Anna V. Scott, Graham N. Stone, Ian P. Vaughan, Jane Memmott

**Affiliations:** 1 School of Biological Sciences, University of Bristol, Life Sciences Building, Bristol BS8 1TQ, UK; 2 Cabot Institute, University of Bristol, Bristol BS8 1UJ, UK; 3 School of Biology, University of Leeds, Leeds LS2 9JT, UK; 4 School of Civil Engineering and Geosciences, Newcastle University, Newcastle upon Tyne NE1 7RU, UK; 5 Institute of Evolutionary Biology, University of Edinburgh, Kings Buildings, Edinburgh EH9 3JT, UK; 6 School of Agriculture, Policy and Development, University of Reading, Reading RG6 6AR, UK; 7 Cardiff School of Biosciences, Cardiff University, Museum Avenue, Cardiff CF10 3AX, UK

**Keywords:** pollinators, networks, urban

## Abstract

Insect pollinators provide a crucial ecosystem service, but are under threat. Urban areas could be important for pollinators, though their value relative to other habitats is poorly known. We compared pollinator communities using quantified flower-visitation networks in 36 sites (each 1 km^2^) in three landscapes: urban, farmland and nature reserves. Overall, flower-visitor abundance and species richness did not differ significantly between the three landscape types. Bee abundance did not differ between landscapes, but bee species richness was higher in urban areas than farmland. Hoverfly abundance was higher in farmland and nature reserves than urban sites, but species richness did not differ significantly. While urban pollinator assemblages were more homogeneous across space than those in farmland or nature reserves, there was no significant difference in the numbers of rarer species between the three landscapes. Network-level specialization was higher in farmland than urban sites. Relative to other habitats, urban visitors foraged from a greater number of plant species (higher generality) but also visited a lower proportion of available plant species (higher specialization), both possibly driven by higher urban plant richness. Urban areas are growing, and improving their value for pollinators should be part of any national strategy to conserve and restore pollinators.

## 1. Introduction

Animal pollination is essential for reproduction in many plant species [1,2] and has been valued globally at €153 billion p.a. (2005) [3] and at more than £510 million p.a. for UK crop production (2009) [4]. However, declines have been reported for all key insect pollinator groups, including honeybees, bumblebees, solitary bees and hoverflies [5–8]. Habitat loss and fragmentation (including urbanization), pesticides, pathogens and their interactions are all proposed drivers of pollinator decline [9,10].

Pollinators have been widely studied in agricultural systems and natural habitats, but urban areas remain under-studied and their suitability for pollinators is unclear. Urbanization represents a major proposed cause of insect decline [11], particularly through alteration of ecological features important to pollinators, such as food and nesting sites [12,13]; many previous studies have found a decrease in the species richness of pollinating insects with increased urbanization (e.g. [14,15]), a trend mirrored in many other animal groups [16,17]. However, urban habitats can contain remarkably high pollinator species richness; for example, 35% of UK hoverfly species were recorded in a single garden [18], half of the German bee fauna has been recorded in Berlin [19], and some studies show a positive effect of urbanization on certain bee taxa, including bumblebees [20] and cavity-nesting bees [21,22]. Urbanization can also change community composition through novel combinations of available species [23], and communities may shift from more specialized to more generalist species [24,25].

Urban land is expanding in the UK [26] and Europe [27], and in 2008 the global proportion of people living in urban areas crossed the 50% threshold [28]. Here, we undertake the first systematic survey of pollinators across the three main land use types in the UK, comparing plant-pollinator communities in thirty-six 1 km^2^ sites in urban areas, farmed landscapes and nature reserves (defined here as land with protected status). We used quantified flower-visitation networks to address three objectives. (i) To compare the abundance, species richness and diversity of insect flower-visitors among the three landscapes. We predict that all measures will be highest in nature reserves and lowest in the urban areas, as previous studies have shown negative impacts of urbanization on insect species richness and abundance [13,29], and intensive agriculture can negatively affect pollinating insects [30,31]. (ii) To compare the composition of insect flower-visitor communities among landscapes. We predict that urbanization will filter out habitat specialists and rare species (e.g. [24]). (iii) To compare insect flower-visitation patterns in urban habitats with those in farmland and in nature reserves. Given that cities often support higher plant species richness [16], we predict that urban pollinators will visit more plant species than their counterparts in other habitats and thus be more generalized in diet.

## 2. Material and methods

### (a) Field site selection

The 36 sites were located in and around 12 large UK urban centres (10 cities and two large towns, all termed cities hereafter) with populations over 150 000. Cities were blocked into four regional groups of three (for city list and map, and selection details, see electronic supplementary material, appendix S1). In each city, we selected a site triplet comprising one urban, one farmland and one nature reserve site. Urban sites were located within the respective city boundary, with matched farmland and nature reserve sites within 10 km of the city boundary. Nature reserve sites were located in National Nature Reserves, Local Nature Reserves or Sites of Special Scientific Interest. Sites were selected using GIS, such that the proportion of habitat types in each site matched those found in the surrounding city, farmland or nature reserve (for full details of methods see electronic supplementary material, appendix S1). All except three of the 36 sites were 100 ha in size; the exceptions were the Edinburgh triplet, in which restrictions on the size of available nature reserves resulted in the selection of 75 ha sites.

### (b) Sampling flowers, flower-visitors and flower-visitor interactions

Each of the 36 sites was sampled four times between 30 May and 19 September 2011 at approximately monthly intervals. Plants and pollinators were sampled along a 2 m × 1 km transect in each site, with sections allocated proportionately to all habitat types comprising more than 1% of the selected site (e.g. pasture, crops, hedgerow and woodland on the farm sites; see electronic supplementary material, appendix S1 for a full list of habitat types). Transects in residential areas were positioned along the boundary between pavements and residential gardens, so that 1 m of the transect width was located in gardens and the other 1 m of the transect width on pavements and road verges. See electronic supplementary material, appendix S1 for further details of site and transect selection.

Flowers were sampled at 10 m intervals along each transect. All flowering plant species in a 0.5 × 0.5 m quadrat were identified and the number of floral units (defined as an individual flower or collection of flowers that an insect of 0.5 cm body length could walk within or fly between) counted for each species. A floral unit comprised a single capitulum for Asteraceae, a secondary umbel for Apiaceae and a single flower for most other taxa (see electronic supplementary material, appendix S2 for full details). Grasses, sedges and wind-pollinated forbs were not sampled.

Flower-visitor interactions were quantified by walking along each transect and collecting all insects (except thrips, order Thysanoptera) on flowers up to 1 m either side of the transect line to a height of 2 m. Each transect was walked twice with a 10-min gap between the two samples to allow disturbed flower-visitors to return. All insects were identified by taxonomists (see Acknowledgements), 95% to species and the remainder to morphologically distinct genera or families. The plant species from which each insect was sampled was identified, 88% to species and the remainder to genus. Sampling for flower-visitors and their interactions took place between 09.00 and 17.00 h on dry, warm, non-windy days spanning the activity periods of diurnally active UK pollinators [32].

### (c) Data analysis

All analyses were performed using R v. 3.1.1 [33]. Generalized linear mixed models (GLMM) were fitted using the R package lme4 [34], with a Gaussian error distribution unless otherwise stated. *Post hoc* Tukey tests were conducted using the multcomp package [35]. The effect of landscape type on the response variable was tested using a log-likelihood ratio test [36] comparing models with and without landscape type included. The effect of region (Scotland, north England, southwest England/Wales, southeast England) was tested but there was no significant effect for any of the models so the term was not included.

#### (i) Objective 1: comparing the abundance, species richness and diversity of insect flower-visitors in urban areas with those in farmland and nature reserves

We tested for the effect of landscape type on species richness and visitor abundance using GLMMs fitted using a Poisson error distribution and a negative binomial distribution respectively. Model residuals were checked for overdispersion and heteroscedasticity. Fixed effects included landscape type (urban, farmland, nature reserve), sampling month (June, July, August, September), floral abundance and proportion of woodland habitat at the site. A nested random effect term of sampling site nested within city was included to reflect the repeated measures of three sites per city. Woodland cover varied greatly among sites, particularly nature reserves, in which it covered 0–96% of site area. Woodland cover was significantly correlated with visitor abundance and therefore included in the model to account for woodland variation across sites. Flower-visitor abundance was included as a covariate in models comparing species richness to control for sample size effects. Analyses were carried out for (i) the whole dataset; (ii) separately for the two dominant insect orders, Diptera and Hymenoptera; (iii) for the key pollinator taxa of hoverflies (Diptera: Syrphidae) and bees (Apoidea: comprising bumblebees, honeybees and solitary bees); and (iv) separately for bumblebees, honeybees and solitary bees. Pollen beetles (Nitidulidae: *Brassicogethes, Kateretes* or *Brachypterus*) were excluded from analyses as they were not observed to move between flowers. Ants (Hymenoptera: Formicidae) and true bugs (Hemiptera) were also excluded as both are considered unimportant as pollinators in the UK [37].

Visitor diversity was calculated for each site using the inverse Simpson's index and Fisher's alpha index [38] as both are relatively robust to differences in sample size. Since Fisher's alpha index could not be calculated for some months at some sites owing to low visitor diversity both indices were calculated for data pooled across months at each site. GLMMs were used to test for differences in diversity between the three landscape types. Models contained landscape type, floral abundance and proportion of woodland as fixed effects, and city as a random effect term to reflect the nested structure of the dataset.

#### (ii) Objective 2: comparing flower-visitor community composition across landscape types

To test if urbanization filters out rare species, we first pooled all of the data from the 36 sites and classified the visitor taxa into four categories based on their overall abundance: (i) more than 100 individuals, (ii) 21–99 individuals, (iii) 2–20 individuals and (iv) 1 individual. While these ranges are arbitrary, they encapsulate the range in abundance from common to rare. We counted the number of recorded taxa per category in each landscape to examine whether rarer species were more frequently found in particular landscape types across our whole dataset. We then recalibrated the categories to grade abundance for each triplet of sites per city so that categories reflected locally common or rare taxa: (i) more than 50 individuals, (ii) 11–49 individuals, (iii) 2–10 individuals and (iv) 1 individual recorded across all sites. We tested whether rare species (those in categories (iii) and (iv)) were found more often in farmland and nature reserve sites than in urban sites using GLMMs fitting a Poisson error distribution. Fixed effects included landscape type, floral abundance and proportion of woodland. Flower-visitor abundance was included as a covariate to control for sample size effects. The random effect term of city was included to reflect the nested structure of the dataset.

Three measures were used to assess similarity in flower-visitor community composition among the 12 sites for each landscape type: (i) Sørensen similarity index to compare species presence/absence between sites; (ii) proportional similarity; and (iii) Horn–Morisita dissimilarity index (see electronic supplementary material, appendix S3 for calculations). The latter two measures incorporate species' relative abundances and both were used as the Horn–Morisita index is independent of sample size but at the cost of being insensitive to turnover in rare species. For the Sørensen index and proportional similarity, a higher value indicates greater similarity whereas a higher Horn–Morisita index indicates lower similarity.

For each site and index, we calculated a mean value over all 11 pairwise comparisons with other sites of the same landscape type, and compared across landscape types using GLMMs, applying the logit transformation for proportions to index values to meet model assumptions. Models included landscape type, floral abundance and proportion of woodland as fixed effects, and city as a random effect term to reflect the nested structure of the dataset. Finally, we visualized variation in community composition across the 36 sites using non-metric multi-dimensional scaling (NMDS) in the R package vegan [38], in which more similar communities group more closely together.

#### (iii) Objective 3: comparing visitor and plant generalization in flower-visitor networks across landscape types

The flower-visitor interaction data were used to construct a flower-visitor network for each of the 36 sites; data were pooled across sampling months for analyses. The R package bipartite [39] was used to calculate the following metrics to enable examination of variation in plant and visitor specialization/generalization across landscape types: ‘generality’, ‘vulnerability’, d′ (species-level specialization) and H2′ (network-level specialization). ‘Generality’ and ‘vulnerability’ were defined by Tylianakis *et al.* [40] in the context of antagonistic plant–parasitoid networks, and here we refer to them as ‘visitor generality’ and ‘plant generality’, respectively. Both are measures of the number of interacting partner species weighted by relative abundance. The d′ metric of specialization measures how specialized a species is with respect to available resources and H2′ represents the overall level of specialization of all species in a network [41]. All metrics were calculated using marginal totals (number of visits per plant species) rather than floral abundance data as the latter were not available for all plant species visited per network (as floral abundance was sampled at 10 m intervals along each transect). Abundances and marginal totals were significantly correlated for plant species with floral abundance data, thus using marginal totals was deemed appropriate. Mean d′ was calculated for (i) plants and (ii) visitors in each network. These five measures (plant generality, visitor generality, mean plant specialization, mean visitor specialization and network-level specialization) were compared across landscape types using GLMMs including the fixed effects landscape type, floral abundance and proportion of woodland, and city as a random effect. Plant and visitor generality were log-transformed and the other response variables logit-transformed to meet model assumptions. d′ and H2′ could not be calculated for the Sheffield nature reserve site as only one plant species (*Calluna vulgaris*) was visited, so the Sheffield site triplet was excluded from these three analyses.

Finally, we compared flowering plant species richness (overall, native and non-native) and numbers of visits to native and non-native plant species between the three landscape types using GLMMs fitted with a Poisson error distribution. Plants were categorized as native or non-native to the British Isles following Hill *et al.* [42]. Models included landscape type, floral abundance and proportion of woodland as fixed effects, and the random effect term of site nested within city.

## 3. Results

Excluding pollen beetles, ants and Hemiptera, a total of 7412 insect flower-visitors were sampled from the 36 sites, of which 67% were Diptera, 26% Hymenoptera, 5% Coleoptera and 2% Lepidoptera. This comprised 412 visitor taxa (262 Diptera, 67 Hymenoptera, 53 Coleoptera and 30 Lepidoptera) visiting 250 plant taxa, and there were 2025 unique interactions between the two groups. Of the 412 visitor taxa, 94% were distinct species or morpho-species and the remainder genus- or family-level identifications.

### (a) Objective 1: comparing the abundance and species richness of insect flower-visitors in urban areas with those in farmland and nature reserves

**Figure 1.**
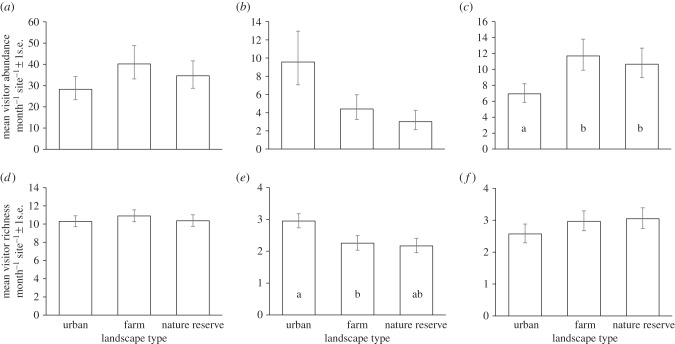
Mean (*a–c*) flower-visitor abundance and (*d–f*) visitor species richness per month per site ± 1 s.e. across the 12 cities for the three landscape types (urban, farmland and nature reserves). Landscape types significantly different from one another are indicated by different letters. Marginal (adjusted) means from the GLMMs, back-transformed to the original scale, are plotted, with standard errors based on the posterior distributions of the regression coefficients using a simulation approach implemented with the R package arm [43]. Results are shown for (*a,d*) all visitors combined, (*b,e*) bees and (*c,f*) hoverflies. Full GLMM results for all taxa are given in table 1.

Summed across all sites, flower-visitors were more abundant in nature reserves (3123) than farmland (2671) and urban sites (1618). Although mean numbers of flower-visitors per site at nature reserve and farmland sites were almost double those at urban sites, there was no significant difference in flower-visitor numbers between the three landscape types (figure 1*a* and table 1). Similarly, overall species richness for the 12 urban sites combined (147) was much lower than for all nature reserves combined (266), or all farmland sites combined (262), but there was no significant difference in the mean visitor species richness or visitor diversity between landscape types (figure 1*d* and table 1).

**Table 1.** Results of GLMMs testing for differences in flower-visitor abundance, species richness and diversity between the three landscape types. Significant results are indicated in bold and there were 2 d.f. for all analyses. Means and standard errors presented are calculated from the raw data and are calculated across the pooled data (i.e. all months combined) for each site, allowing direct comparisons between abundance and richness, where monthly variation was modelled in the GLMMs, and diversity, where GLMMs pooled data across months. Significant *post hoc* Tukey tests used to test for differences between landscape pairs are shown, near-significant *p*-values are given in brackets and all other pairwise comparisons were not significant. UR, urban; FM, farmland; NR, nature reserve sites.

	mean abundance, richness or diversity ± 1 s.e. across sites for all months combined			effect of landscape type		Tukey *post hoc* tests	
taxon or index	urban	farmland	nature reserve	*χ*^2^	*p*-value	direction	*p*-value
*visitor abundance*							
all taxa	134.83 ± 17.31	222.58 ± 43.80	260.25 ± 65.74	5.405	(0.067)	NR > UR	(0.057)
Hymenoptera	64.58 ± 12.65	51.08 ± 12.89	45.83 ± 15.31	1.575	0.455	—	—
bees	54.83 ± 11.53	45.08 ± 13.31	41.25 ± 15.00	1.315	0.518	—	—
bumblebees	34.42 ± 4.96	25.58 ± 7.57	28.75 ± 13.51	3.052	0.217	—	—
honeybees	14.50 ± 6.39	16.83 ± 5.80	10.50 ± 4.24	0.396	0.820	—	—
solitary bees	5.92 ± 2.19	4.75 ± 1.96	2.00 ± 1.02	0.863	0.650	—	—
Diptera	62.67 ± 12.03	157.83 ± 40.61	192.75 ± 50.72	12.138	**0.002**	FM > UR NR > UR	**0.003** **0.002**
hoverflies	43.42 ± 9.36	57.42 ± 12.77	94.08 ± 35.18	8.228	**0.016**	FM > UR NR > UR	**0.025** **0.021**
*visitor richness*							
all taxa	31.67 ± 3.58	48.25 ± 7.00	46.25 ± 8.73	0.638	0.727	—	—
Hymenoptera	11.33 ± 1.45	9.92 ± 1.28	9.00 ± 1.31	2.453	0.293	—	—
bees	9.33 ± 1.20	7.25 ± 1.09	6.25 ± 0.83	6.459	**0.040**	FM < UR NR < UR	**0.049** (0.053)
bumblebees	5.00 ± 0.49	4.00 ± 0.52	4.58 ± 0.62	4.177	0.124	—	—
solitary bees	3.42 ± 0.99	2.50 ± 0.83	1.00 ± 0.35	1.268	0.531	—	—
Diptera	17.75 ± 2.16	32.42 ± 5.75	30.33 ± 6.46	1.809	0.405	—	—
hoverflies	8.67 ± 0.83	12.17 ± 1.93	12.42 ± 2.19	1.956	0.376	—	—
*visitor diversity*							
inverse Simpson's	8.21 ± 1.14	10.63 ± 1.07	10.79 ± 2.03	2.439	0.295	—	—
Fisher's *α*	14.87 ± 2.14	20.08 ± 2.05	17.90 ± 2.90	5.762	0.056	FM > UR	(0.063)

Hymenopteran abundance and species richness were not significantly different between landscape types (table 1). Bees contributed most hymenopteran visits (90%), with solitary bees, bumblebees and honeybees contributing 9%, 62% and 29% of bee visits, respectively. For bees alone, while overall abundance did not differ significantly among landscape types, bee species richness in urban landscapes was significantly higher than in farmland, and approaching significance for nature reserves (*p* = 0.053; figure 1*b,e* and table 1). Separate analyses for honeybees, bumblebees and solitary bees showed no significant differences in richness or abundance among landscape types (table 1).

Dipteran abundance was significantly higher in farmland and nature reserves than in urban sites, although there were no differences in richness (table 1). More specifically, hoverflies (Syrphidae) contributed a greater proportion of dipteran flower visits in urban sites (69%) than in farmland (36%) and nature reserves (49%). There were significantly more hoverflies in farmland and nature reserve sites than in urban areas (table 1 and figure 1*c*), although hoverfly species richness did not differ among the three landscapes (figure 1*f* and table 1). The net effect is that while urban sites have fewer flies, their dipteran assemblage is enriched in hoverflies relative to farms and nature reserves.

### (b) Objective 2: comparing flower-visitor community composition across landscape types

**Figure 2.**
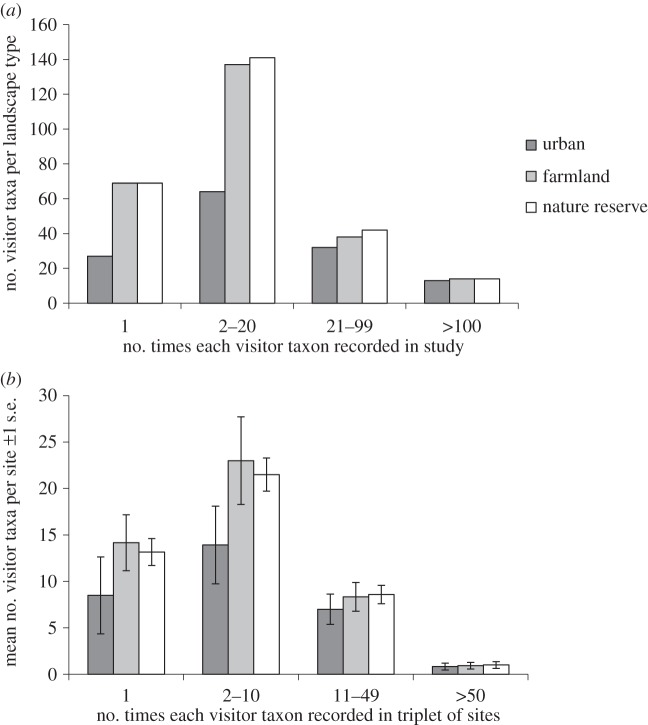
Numbers of rare, intermediate and common visitor taxa found in (*a*) the whole dataset and (*b*) individual sites. Urban sites are shown in dark grey, farmland sites in light grey and nature reserves in white.

When sites of each landscape type were combined and rarity categories assigned at a national scale, rare taxa were more often found in nature reserve and farmland than in urban sites (figure 2*a*; electronic supplementary material, appendix S4). When rarity categories were assigned at a local scale (i.e. within a triplet), there was no significant difference between landscape types in the number of rare taxa recorded and they made up a similar proportion of visitor taxa for all three landscape types (figure 2*b*; electronic supplementary material, appendix S4). Eleven flower-visitor species classified as nationally rare or scarce [44,45] were found, four of them in urban sites (electronic supplementary material, appendix S5).

Overall, flower-visitor communities in urban areas were more homogeneous across sites than were those from nature reserve or farmland sites (electronic supplementary material, appendix S6). Both mean Sørensen and mean proportional similarity indices were significantly higher for urban sites than for farmland and nature reserves (table 2). Mean Horn–Morisita indices (a dissimilarity index) were significantly lower in urban than farmland sites, although not lower than in nature reserves (*p* = 0.09 for the latter comparison; table 2), consistent with greater visitor community similarity among urban sites than among farmland sites.

**Table 2.** Results of GLMMs testing for differences in flower-visitor community composition between the three landscape types. Significant results are indicated in bold and there were 2 d.f. for all analyses. Means and standard errors are calculated from the raw data. Significant *post hoc* Tukey tests used to test for differences between landscape pairs are shown, near-significant *p*-values are given in brackets and all other pairwise comparisons were not significant. UR, urban; FM, farmland; NR, nature reserve sites.

	mean index value ± 1 s.e.			effect of landscape type		Tukey *post hoc* tests	
index	urban	farmland	nature reserve	*χ*^2^	*p*-value	direction	*p*-value
Sørensen similarity index	0.370 ± 0.018	0.272 ± 0.016	0.246 ± 0.010	20.741	**<0.0001**	FM < UR NR < UR	<**0.0001** <**0.0001**
proportional similarity	0.356 ± 0.024	0.247 ± 0.013	0.234 ± 0.016	24.747	<**0.0001**	FM < UR NR < UR	< **0.0001** <**0.0001**
Horn–Morisita dissimilarity index	0.531 ± 0.038	0.644 ± 0.027	0.664 ± 0.033	7.529	**0.023**	FM > UR NR > UR	**0.030** (0.0901)

### (c) Objective 3: comparing visitor and plant generalization in flower-visitor networks across landscape types

**Figure 3.**
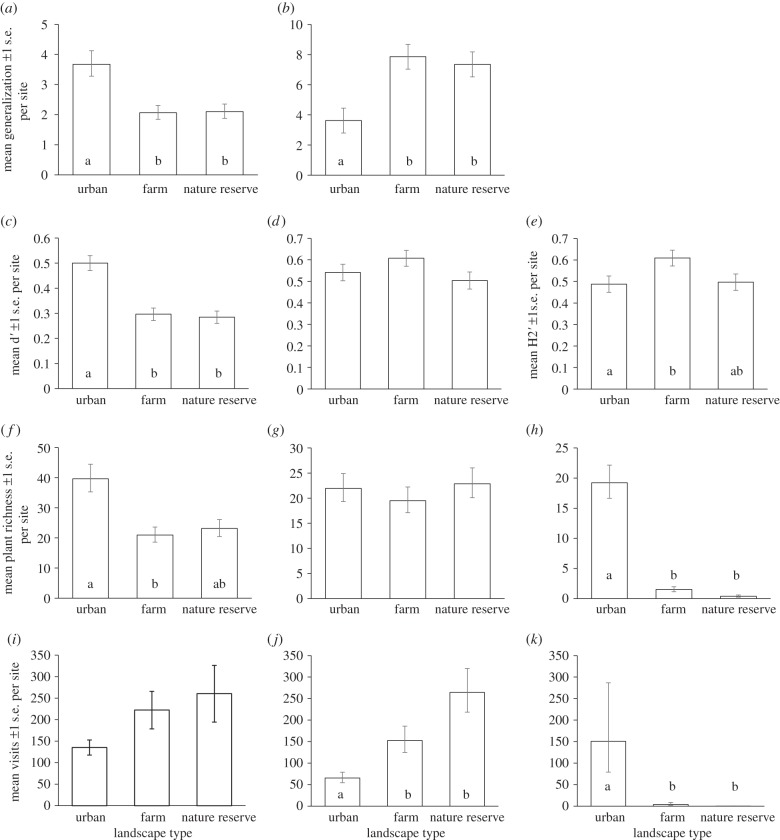
Mean site-level values ± 1 s.e. for (*a*) visitor generality, (*b*) plant generality, (*c*) visitor specialization (d′), (*d*) plant specialization (d′), (*e*) network specialization (H2′), (*f*) flowering plant richness, (*g*) native flowering plant richness, (*h*) non-native flowering plant richness, (*i*) total flower visits, (*j*) native flower visits and (*k*) non-native flower visits. Landscape types significantly different from one another are indicated by different letters. Full GLMM results are given in electronic supplementary material, appendix S7. Marginal (adjusted) means from the GLMMs, back-transformed to the original scale, are plotted and standard errors based on the posterior distributions of the regression coefficients using a simulation approach implemented with the R package arm [43].

Visitor generality (in terms of numbers of plant species visited) was significantly higher in urban compared with farmland and nature reserve sites (figure 3*a*; electronic supplementary material, appendix S7), with visitor taxa in urban sites visiting more plant species on average than those in other sites. Conversely, plant generality (in terms of numbers of visitor taxa) was significantly lower at urban sites than in farmland and nature reserves (figure 3*b*); thus plant species in farmland and nature reserve sites received visits from a greater variety of visitor taxa than those in urban areas. Mean visitor species-level specialization was significantly higher in urban sites compared with farmland and nature reserve sites (figure 3*c*), which indicates that visitors in urban areas made use of a smaller fraction of the available floral resources. There was no significant difference in plant species-level specialization between landscape types (figure 3*d*). Network-level specialization, which combines plants and visitors, and thus examines interaction-level specialization, was significantly higher in farmland than urban sites (figure 3*e*).

Plant species richness was significantly higher in urban areas than farmland (figure 3*f*), an effect driven by higher richness of non-native plants: while native plant species richness was not different between the three landscapes, there were significantly more non-native plant species in urban areas (figures 3*g,h*). Similar numbers of visits were recorded to native and non-native plant species in urban sites; by contrast, almost all flower-visitors were recorded on native plant species in farmland and nature reserve sites (figure 3*j,k*).

## 4. Discussion

This is the first study to systematically compare pollinator communities in replicate urban and non-urban landscapes; moreover, it is based on highly resolved flower–visitor interaction networks. Our results show that while there was no difference in pollinator abundance and richness between urban, farmland and nature reserve sites, patterns varied between taxa. Bee species richness was higher and flies were less abundant in urban areas, as were hoverflies when considered separately. Urban areas had more homogeneous visitor communities than farmland or nature reserves, although they contained similar numbers of rare flower-visitor taxa. In what follows, we first address limitations of our study and then discuss our results, first in the context of our objectives, and then in the wider context of urban ecology and conservation management.

### (a) Limitations

There are two main limitations to our work. First, because sampling started in late May some early spring solitary bees are likely to have been missed, especially at southern sites. However, our sampling was not designed to survey each site exhaustively; rather, we aimed to sample multiple sites regularly through the year using a standardized approach to make broad cross-landscape comparisons. Second, using transect sampling rather than targeted observations of each flowering plant species probably missed some rare pollinator taxa [46]. Transects, nevertheless, allow efficient sampling of many sites under time constraints [46]. Furthermore, the high plant species richness at urban sites would have resulted in a much higher sampling effort at urban sites if data had been gathered using timed observations per plant species. All insect sampling methods suffer from a variety of biases [47], and overall transect samples were deemed the most appropriate approach for this study.

### (b) Objective 1: comparing the abundance and species richness of insect flower-visitors in urban areas with those in farmland and nature reserves

Other studies comparing potential pollinators between urban and non-urban habitats have found a negative effect of urbanization on the abundance and species richness of flower-visiting insects [12,13,15]. Although our study found no significant differences in overall abundance or richness among urban, farmland and nature reserve habitats for all visitor taxa combined, our results suggest that numbers of fly and hoverfly visitors were higher in non-urban compared with urban habitats. Deguines *et al.* [13] found urbanization to have a lesser effect on bees than on other insects, a result mirrored in our data with higher bee species richness in urban compared with farmland sites. Bees require two main resources: food (generally in the forms of pollen and nectar) and a suitable nesting site. Food in urban areas is provided by a combination of native and introduced plant species. Although some horticultural plant varieties may not provide as much pollen or nectar as non-modified varieties (e.g. single versus double flowers [48]), many non-native plants can provide large quantities of both rewards [49]. Bees nest in a variety of locations, including soil, pre-existing cavities in walls and other structures, pithy plant stems and trees, and heterogeneous urban habitats can provide suitable nesting sites for a wide range of bee taxa [50].

Our results show that abundance and richness were no different for farmland compared with nature reserves for any of the visitor taxa. One explanation for our findings could be high habitat heterogeneity between the different nature reserves sampled, which ranged from woodland to meadow to heathland. These sites showed large differences in floral communities and flowering phenologies, and while some nature reserve sites were very good for pollinators, others, particularly woodland-dominated sites in southern England, had very few flower-visitors during our sampling period. Although all reserve sites had protected status, they were not designated on the basis of their suitability for pollinators.

### (c) Objective 2: comparing the composition of urban flower-visitor communities with farmland and nature reserves

There was no difference in the number of rarer taxa in our dataset among urban, farmland and nature reserve sites. However, we recognize that visitor taxa classified as ‘rare’ in our dataset may not reflect their overall status. We recorded three species designated as priority species according to the UK Biodiversity Action Plan. *Bombus humilis* was recorded in the Cardiff urban, farmland and nature reserve sites, and two rare butterflies (*Boloria selene* and *Coenonympha pamphilus*) at nature reserve sites (electronic supplementary material, appendix S5). Hoverfly species noted as nationally scarce [45] and bee taxa noted as scarce or threatened [44] were also recorded in all three landscapes (electronic supplementary material, appendix S5). Our findings suggest that urban areas contain lower overall species richness across the wider landscape (although bee richness is comparatively high) and contain somewhat homogeneous visitor assemblages. While previous studies suggest urban areas contain fewer habitat specialists and rare species (e.g. [14]), our findings suggest that the differences between urban and non-urban habitats may not be large with respect to rare species.

### (d) Objective 3: comparing visitor and plant generalization in urban flower-visitor networks with farmland and nature reserves

While visitors were recorded on more plant species in urban areas, they also visited a lower proportion of the plant species available compared with non-urban sites. This generates the apparently contrasting patterns in visitor generality (number of plants visited) and specialization (proportion of available plant species visited). These findings probably reflect the much higher richness of flowering plant species, driven by higher non-native richness, in urban areas. The greater generalization of urban visitors could potentially render them less effective pollinators as they are likely to be carrying more pollen species [51]. Conversely, plant generality was higher in non-urban habitats; plants were on average visited by more visitor taxa in farmland and nature reserve habitats. This can be explained by the lower plant species richness in non-urban habitats, meaning that visitor taxa had fewer plant species to visit. Overall, interactions at farmland sites were more specialized than those in urban areas, a result probably also driven by lower plant richness.

### (e) Conclusion and future directions

This is the first study to compare pollinator communities in urban and non-urban habitats with replication across multiple geographically separate urban locations. Our findings suggest that urban areas can contain high bee species richness, although hoverfly abundance was lower in urban areas than elsewhere. While the effects of urbanization are likely to differ between regions and climates depending on the composition of the local pollinator fauna, urban areas are expanding globally, and natural and semi-natural habitats that support pollinator populations are likely to decrease. If high-quality urban areas are able to support good populations of insect pollinators, they could act as important source areas, refuges and corridors of favourable habitat in a hostile matrix habitat such as intensive agricultural landscapes. While there has been increasing interest in enhancing agricultural areas for pollinators, far less attention has been paid to how urban areas can be made more pollinator-friendly. Given the fact that urban areas are widespread and that there are likely to be increasing pressures on more natural areas for food and biofuel production, identifying good urban habitats for pollinators and improving their value for pollinators should be part of any strategy to conserve and restore pollinators.

## Supplementary Material

Electronic Supplementary Material: All files

## Supplementary Material

Datasets

## Data accessibility

The datasets used in analyses are included as electronic supplementary material.
