# Supplementary material for: Where is the UK's pollinator biodiversity? The importance of urban areas for flower-visiting insects
Source: Proc Biol Sci. 2015 Mar 22;282(1803):20142849. doi: 10.1098/rspb.2014.2849 (PMC4345454; doi:10.1098/rspb.2014.2849)
Supplement: Electronic Supplementary Material: All files [file rspb20142849supp1.docx]

**Electronic Supplementary Material**

**Appendix 1. Site selection**

**Part A Selection of the 12 urban centres**

1. Population data were downloaded for each town and city in the UK (Office for National Statistics 2011a, General Register Office for Scotland 2011). A series of ‘urban centres’ were selected if their population was over 150,000.
2. The host cities of Reading, Bristol, Edinburgh and Leeds, which span north, south, east and west of the UK, were used as starting points for the selection of other cities. Each host city has a population of over 150,000 and so was included.
3. Further study sites needed to be within travelling distance of the host cities, but far enough away that they could be considered to contribute statistically independent landscape samples. Therefore towns or cities were selected if they were more than 25 km, but less than 100 km from host cities. If more than two cities were available using these methods then a final set was selected based upon practical and logistical considerations. For practical purposes Greater London was taken as being one urban centre. The 12 towns and cities (all termed ‘cities’ hereafter) used are listed in Table 1.1 and their UK distribution is mapped in Figure 1.1.

**Table 1.1 The 12 cities used in the study**

| **City** | **Region** |
| --- | --- |
| Bristol | SW England/Wales |
| Cardiff | SW England/Wales |
| Swindon | SW England/Wales |
| Reading (includes adjacent urban area of Wokingham) | SE England |
| Greater London | SE England |
| Southampton | SE England |
| Leeds | N England |
| Sheffield | N England |
| Kingston-upon-Hull | N England |
| Edinburgh | Scotland |
| Glasgow | Scotland |
| Dundee | Scotland |

**
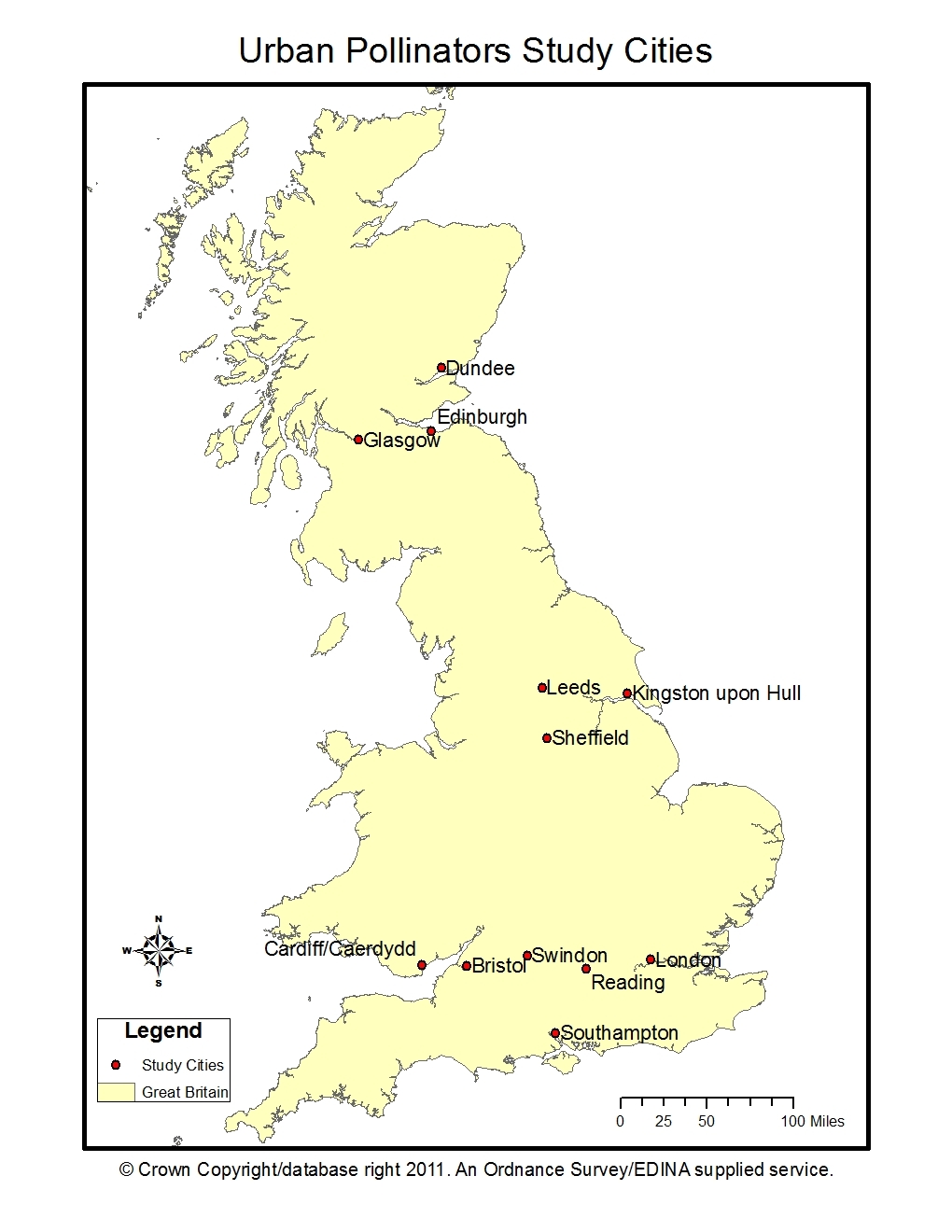
**

**Figure 1.1 Locations of the twelve cities used for sampling**

A triplet of sites (one urban, one farmland and one nature reserve) was located in and around each city.

**Part B Selection of the 36 sampling sites**

## Creation of selection zones for each urban area

## Datasets of urban settlements in England, Scotland and Wales were downloaded to show the urban region of each study city (Office for National Statistics 2011b, National Records of Scotland 2011). These datasets were used to define the urban zone for each study city.

## A buffer of 10km was created around each urban zone. This buffer was clipped so that all ‘urbanised’ areas were removed – this included satellite towns and villages that were not included in the urban zone of the main study city. This was then used as the buffer zone for selecting farmland and nature reserve sites.

## Identification of potential urban and farmland sampling sites

- 1. Land Cover Map 2000 data were obtained in a raster format (Natural Environment Research Council 2000). For each zone (i.e. urban or buffer) the total area of each land cover type was calculated from the LCM2000 data using Hawth’s tools (Beyer 2004) and ArcGIS 9.3.
  2. Four land cover types were removed from the analysis: Sea/Estuary, Water (inland), Littoral Rock, and Littoral Sediment. These land cover types were removed because they are dangerous and impractical to sample, and they are very unlikely to support pollinator populations. The proportion of each remaining land cover type within the urban and buffer zone of each study city was calculated.
  3. The urban and buffer zone of each study city was divided into 1000 x 1000 m squares based on the British National Grid. The proportion of land covers within each 1 km square was calculated using Hawth’s tools in ArcGIS 9.3.
  4. The total proportions that had previously been calculated for each zone were used as a guide for selecting individual 1 km squares that had similar (+/-8%) proportions of land cover types to the whole of the zone in which they were located. This was done using the ‘select by attributes’ tool in ArcGIS. Habitats were only selected for if they made up 5% or more of the total land cover in the respective zone.
  5. Thus for each urban and buffer zone, two shortlists of potential sites were created: (i) individual 1 km squares within the urban zone that were representative of the land cover types within the whole of the urban zone and (ii) individual 1 km squares within the buffer zone that were representative of the land cover types within the whole of the buffer zone. 1 km squares within the urban zone represented potential urban sites and 1 km squares within the buffer zone represented potential farmland sites. For the Edinburgh sampling sites an identical process was repeated with grid squares of size 0.87 x 0.87 km with an area of 0.75 km^2^ so that urban and farmland sites were the same size as the only available nature reserve site (see Section 6.1).

1. **Selection of urban sampling sites**
   1. For each city, one of the shortlisted squares in the urban zone was chosen as that city’s urban site. The square selected was the urban square that could be most easily and safely accessed from the host institution.
2. **Selection of farmland sampling sites**
   1. For each city, one of the shortlisted squares in the buffer zone was chosen as that city’s farmland site. Squares which were <500 m from the urban zone and squares which contained <70% farmland were removed from the shortlist. LCM habitat categories classed as representative of farmland were ‘Cereals’, ‘Horticulture/non-cereal or unknown’, ‘Not annual crop’ and all grassland categories. Squares for which these categories formed >70% of the total area were examined using Google Earth to confirm that farmland accounted for >70% of the square.
   2. The square with the shortest travel time from the host institution was selected in order to minimise travel time for fieldwork. If the selected square was <2 km from the nature reserve or urban sampling site it was not used and the next closest square to the host institution selected until this criterion could be fulfilled. If permission could not be obtained from landowners to sample the selected square, the next closest square to the host institution selected until this criterion could be fulfilled.
3. **Selection of nature reserve sites**
   1. The location of nature reserves (NNR, LNR, SSSI, SPA, SAC, Ramsar) were downloaded from Natural England (2011), CCW (2011) and Scottish Natural Heritage ([2011](http://gateway.snh.gov.uk)). Nature reserves designated for geological features rather than ecological features were excluded. The eighteen sources of data were combined into one layer. They were joined in the following order, with the first taking precedence over the next: LNR > NNR > SSSI > SPA > SAC > Ramsar.
   2. The polygons were dissolved without creating multi-part features, meaning that polygons with the same name whose boundaries touched were treated as one site. However, unless the boundaries touched each polygon was treated as a separate site even if it had the same name as another. This was to remove any issues associated with multipart nature reserves that were some distance apart.
   3. All nature reserves within or partly within the buffer zones of each city were selected.
   4. Nature reserves that were smaller than 70 ha or greater than 600 ha were removed. The smaller nature reserves were removed because the nature reserve had to be comparable to the urban and farmland sites, of a size that would accommodate a 1000 m transect and cover a number of habitats. The larger nature reserves were removed so that there was no bias from having a particularly large nature reserve. As with the urban and farmland sites, any nature reserves which were above 200 m elevation were also removed from the analysis.
   5. The overall proportions of each land cover, as categorised by the LCM2000, were calculated for all shortlisted nature reserves. This was done using the ‘thematic raster summary (by polygon)’ in Hawth’s tools. These were then summed together to produce a list of the most dominant land covers found in nature reserves around each individual city. The final selection of nature reserves was based on: (i) the site being representative of the dominant land cover(s) in nature reserves surrounding that city; (ii) accessibility for fieldwork; and (iii) permission being obtained for sampling.
   6. For nature reserves of 100 ha or close to 100 ha in size, the entire nature reserve was used as a sampling site. For nature reserves greater than 100 ha a rectangular area of 100 ha in size was located at random within the nature reserve.

## 6. Exceptions

1. The sizes of all Edinburgh sites were reduced to 75 ha rather than 100 ha because the largest nature reserve in the area was 75 ha
2. One nature reserve - Fyfield Down NNR/SSSI - was included even though it was 300 m outside the buffer and marginally higher than the 200m altitude limit. This was because the reserve fitted all the other criteria and no other nature reserve sites within the Swindon buffer zone were large enough to be included in the study.
3. In Kingston-upon-Hull two nature reserves (Far Ings NNR/LNR and Water’s Edge LNR) that were adjacent to one another were combined. This was because there were no other reserves large enough to be included in the study, and also because they covered the type of habitat that was dominant in nature reserves around this city.
4. The Sheffield nature reserve site was a 237 ha section of the Eastern Peak District Moors SSSI. Although this site was at a higher elevation than the 200 m altitude limit, it was selected because permission could not be obtained to survey a representative nature reserve at a lower elevation.

**Table 1.2 Datasets and sources**

| Data Description | Source |
| --- | --- |
| English and Welsh Census Data 2001 -Usual resident population | Office for National Statistics (2011a) |
| Scottish Census Data 2001 -Total resident population | General Register Office for Scotland (2011) |
| English and Welsh Urban Areas 2001 | Office for National Statistics (2011b) |
| Scottish Settlements 2001 | National Records of Scotland (2011) |
| Land Cover Map 2000 | Natural Environment Research Council (Centre for Ecology and Hydrology) (2000) |
| English Nature Reserves (LNR, NNR, SSSI, SAC, SPA, Ramsar) | Natural England (2011) |
| Welsh Nature Reserves (LNR, NNR, SSSI, SAC, SPA, Ramsar) | Countryside Council for Wales (2011) |
| Scottish Nature Reserves (LNR, NNR, SSSI, SAC, SPA, Ramsar) | Scottish Natural Heritage (2011) |
| UK STRM Digital Elevation Model | NASA/NGA/DLR/ASI (2011) |

**Table 1.3 The 36 sampling sites used in the study**

| **Urban area** | **Landscape type** | **Site area** | **Site name & designation** | **Dominant NR habitat** |
| --- | --- | --- | --- | --- |
| Bristol | Urban | 1 km^2^ | Westbury-on-Trym |  |
| Bristol | Farmland | 1 km^2^ | Barrow Gurney |  |
| Bristol | nature reserve | 1 km^2^ | Ashton Court SSSI | Grassland/woodland |
| Cardiff | Urban | 1 km^2^ | Heath |  |
| Cardiff | Farmland | 1 km^2^ | Lower Stockland |  |
| Cardiff | nature reserve | 1 km^2^ | Llantrisant Common SSSI | Grassland |
| Dundee | Urban | 1 km^2^ | Victoria Park |  |
| Dundee | Farmland | 1 km^2^ | Brunton |  |
| Dundee | nature reserve | 1 km^2^ | Earlshall Muir SSSI | Grassland but mixed |
| Edinburgh | Urban | 0.75 km^2^ | Morningside |  |
| Edinburgh | Farmland | 0.75 km^2^ | nr Temple, Gorebridge |  |
| Edinburgh | nature reserve | 0.75 km^2^ | Crichton Glen SSSI | Grassland/woodland |
| Glasgow | Urban | 1 km^2^ | Portormin Road |  |
| Glasgow | Farmland | 1 km^2^ | North of Airdrie |  |
| Glasgow | nature reserve | 1 km^2^ | Mugdock Wood SSSI | Broad-leaved woodland |
| Kingston-upon-Hull | Urban | 1 km^2^ | Gipsyville |  |
| Kingston-upon-Hull | Farmland | 1 km^2^ | Rudstone Walk, South Newbald |  |
| Kingston-upon-Hull | nature reserve | 1 km^2^ | Far Ings NNR, LNR, Waters Edge LNR | Mixed grassland, wetland & other |
| Greater London | Urban | 1 km^2^ | Hayes & Harlington |  |
| Greater London | Farmland | 1 km^2^ | Southeast of Potters Bar (Botany Bay) |  |
| Greater London | nature reserve | 1 km^2^ | Burnham Beeches NNR, SSSI, SAC | Broad-leaved woodland |
| Leeds | urban | 1 km^2^ | Headingley/Meanwood |  |
| Leeds | farmland | 1 km^2^ | Harewood |  |
| Leeds | nature reserve | 1 km^2^ | Newmillerdam LNR | Broad-leaved woodland |
| Reading | urban | 1 km^2^ | Loddon Bridge |  |
| Reading | farmland | 1 km^2^ | Farley Hill |  |
| Reading | nature reserve | 1 km^2^ | Bramshill SSSI | Coniferous woodland |
| Sheffield | urban | 1 km^2^ | Wadsley Bridge |  |
| Sheffield | farmland | 1 km^2^ | Hermit Hill |  |
| Sheffield | nature reserve | 1 km^2^ | Eastern Peak District Moors SSSI | Heathland |
| Southampton | urban | 1 km^2^ | Portswood |  |
| Southampton | farmland | 1 km^2^ | South of Braishfield |  |
| Southampton | nature reserve | 1 km^2^ | Botley Wood and Everett’s and Mushes Copses SSSI | Broad-leaved woodland |
| Swindon | urban | 1 km^2^ | Grange Park |  |
| Swindon | farmland | 1 km^2^ | Can Court Farm |  |
| Swindon | nature reserve | 1 km^2^ | Fyfield Down NNR SSSI | Grassland |

**References**

Beyer, H. L. 2004. Hawth's Analysis Tools for ArcGIS. Available at http://www.spatialecology.com/htools.

General Register Office for Scotland 2011. 2001 Census: Population data (Scotland) [Computer file]. Scotland’s Census Results Online. Downloaded from: http://www.scrol.gov.uk/scrol/

Office for National Statistics 2011a. 2001 Census: Population data (England and Wales) [Computer files]. UK Data Service (Casweb). Downloaded from: http://casweb.mimas.ac.uk/

Office for National Statistics 2011b. 2001 Census: Digitised Boundary Data (England and Wales) [computer file]. UK Data Service Census Support (EDINA). Downloaded from: http://edina.ac.uk/census

National Records of Scotland 2011. 2001 Census: Digitised Boundary Data (Scotland) [computer file]. UK Data Service Census Support (EDINA)). Downloaded from: http://edina.ac.uk/census

Natural Environment Research Council (Centre for Ecology and Hydrology) 2000. Land Cover Map 2000 [Computer file]. Downloaded from http://www.ceh.ac.uk/landcovermap2000.html

Natural England 2011. Digitised boundary data (England) [computer file]. Downloaded from http://www.gis.naturalengland.org.uk/pubs/gis/GIS_register.asp

Countryside Council for Wales 2011. Digitised boundary data (Wales) [computer file]. Downloaded from http://www.ccw.gov.uk/landscape--wildlife/protecting-our-landscape/gis-download---welcome/

Scottish Natural Heritage 2011. Digitised boundary data (Scotland) [computer file]. Downloaded from http://gateway.snh.gov.uk/sitelink/

NASA/NGA/DLR/ASI 2011. UK STRM Digital Elevation Model [computer file]. Sourced from ShareGeo (EDINA). Downloaded from http://edina.ac.uk/projects/sharegeo/

**Part C. Transect selection methods and sampling approach**

1. The 36 sampling sites were visited and the habitats detailed in Table 1.4 were mapped for each site. Habitat categories were defined for the project, although farmland habitats followed definitions in Gibson *et al.* (2007).
2. The area of each habitat was calculated for each site by measuring the sizes of individual polygons using Magic Map (http://magic.defra.gov.uk).
3. The proportion of the site covered by each habitat was calculated for each site.
4. A total transect length of 1 km was used at each site. Each transect was 2 m wide. The transect length was divided proportionally between habitat types that comprised more than 1% of the site.
5. Transect locations were chosen at random by using a random number generator to select points at random within each site. The transect was located as close to the random point as possible that would allow a transect of the required distance and habitat to be walked.
6. Where habitats were particularly dominant within a site, a maximum transect distance of 250 m was used to ensure that these habitats were sampled at multiple locations.
7. For each sampling visit (one per month) each transect was walked twice for flower-visitor sampling. There was a gap of at least ten minutes between the two transect walks.
8. The transects at most sites could be sampled in a single day. If a site could not be sampled in a single day, sampling was completed on the next day with suitable weather conditions before moving to sample another site. The same transects were used on each sampling visit within each site, but they were sampled in a different order to reduce bias caused by time of day.

An example of how transects were selected is shown in Figure 1.2 and Table 1.5.

**Table 1.4 Habitats mapped for each of the 36 sampling sites**

|  | **Habitat** | **Habitat Code** | **Description** |
| --- | --- | --- | --- |
| **Urban** | *Residential* | **UR_RES** | Front gardens, pavements, road verges and small patches of amenity grassland within residential areas, shops sharing same building as residential housing. Roads within residential areas were included as residential habitat. |
|  | *Allotments* | **UR_ALT** | Allotments: council owned and private |
|  | *Commercial & Public Buildings* | **UR_COM** | Shopping centres, leisure parks, supermarkets, hospitals, petrol stations, school buildings & associated car parks/roads/paved areas |
|  | *Industrial* | **UR_IND** | Industrial estates, includes buildings, car parks, roads and pavements |
|  | *Amenity grassland* | **UR_AMY** | Large patches of improved grassland receiving high levels of management throughout the year. Includes parks, sports fields, school fields, road verges outside residential areas. Includes scrub and scattered trees present in grassland. Includes paths running through grassland. |
|  | *Rough grassland* | **UR_RGR** | Grassland that is unmanaged or receives infrequent formal management. Includes scrub and scattered trees in grassland. |
|  | *Broadleaved Woodland* | **UR_BLW** | Broad-leaved/mixed woodland |
|  | *Coniferous Woodland* | **UR_CW** | Coniferous woodland |
|  | *Farmland* | **UR_FM** | Land managed for agriculture |
|  | *Other* | **UR_OTH** | Large roads (e.g. dual carriageways) |
| **Farmland** | *Arable* | **FM_ARA** | All crops sown or growing during the survey period (Gibson *et al*. 2007) |
|  | *Grass* | **FM_PAS** | Improved and permanent pastures, and grass leys (Gibson *et al*. 2007) |
|  | *Rough ground* | **FM_RGR** | Land not managed by the farmer in order to return a profit; including land unsuitable for cultivation, dumping areas for farm machinery and animal waste (Gibson *et al*. 2007) |
|  | *Linear boundary habitat* | **FM_LIN** | Hedgerows and field margins.  *Hedgerows*: vegetation thick from the ground up and forming an obvious boundary, or, vegetation thick above waist height with trunks visible below, but forming a think continuous field boundary of even height and width (Gibson *et al*. 2007).  *Field margins*: Semi-natural habitat (uncultivated) > 1 m in width that formed the perimeter of a field and was located between the crop and the fence-line or hedgerow (Gibson *et al*. 2007) |
|  | *Broadleaved Woodland* | **FM_BLW** | Broad-leaved/mixed woodland |
|  | *Coniferous Woodland* | **FM_CW** | Coniferous woodland |
|  | *Other* | **FM_OTH** | Includes farm buildings, farmyard, landfill sites, rural residential areas, road verge and an arboretum site. |
| **Nature**  **Reserve** | *Broadleaved Woodland* | **NR_BLW** | Broad-leaved/mixed woodland |
|  | *Coniferous Woodland* | **NR_CW** | Coniferous woodland |
|  | *Mixed woodland* | **NR_MX** | Mixed woodland |
|  | *Grassland* | **NR_GLD** | All types of grassland. Includes scrub and scattered trees. |
|  | *Heathland* | **NR_HLD** | All types of heathland |
|  | *Wetland* | **NR_WTD** | Any wetland habitat |

Gibson, R, Pearce, S., Morris, R., Symondson, W. & Memmott, J. 2007 Plant diversity and land use under organic and conventional agriculture: a whole-farm approach. *Journal of Applied Ecology* **44** 792 – 803.

**Figure 1.2 An example of transect locations: Swindon urban site** The red 1 km x 1 km square shows the outline of the site. Transect walks are shown as red lines and locations were selected at random. Urban habitats were mapped by field teams: namely residential, woodland, commercial and amenity grassland at this site (see Table 1.4 for habitat definitions). The area covered by each habitat was calculated using Magic Map and the proportion of the site covered by each habitat calculated. The 1 km transect distance for the site was split proportionally between the habitats present (see table 1.5).

**Table 1.5 Habitat areas and transect lengths for each habitat at the Swindon urban site**

| **Habitat** | **Proportion of site** | **Total transect length (m)** |
| --- | --- | --- |
| Amenity grassland | 0.234 | 234 |
| Commercial | 0.143 | 143 |
| Residential | 0.529 | 529 |
| Woodland | 0.094 | 94 |

**Appendix 2. Floral unit definitions**

**Table 2.1 How ‘Floral units’ were defined for all plant taxa sampled in the study**

| **Floral Unit definition** | **Plant taxa** |
| --- | --- |
| Single flower | *Alstroemeria* spp., all Amaranthaceae, *Allium* spp., *Vinca* spp., *Ilex* spp., *Zantedeschia* spp., *Hedera* spp., *Hyacinthoides* spp., *Impatiens* spp., *Berberis* spp., *Mahonia* spp., all Boraginaceae, all Brassicaceae, all Campanulaceae, all Caprifoliaceae (apart from *Sambucus* spp.), all Caryophyllaceae, *Euonymus* spp., all Cistaceae, all Convolvulaceae, *Sedum* spp., *Dipsacus fullonum*, *Eleagnus* spp., all Ericaceae (apart from *Calluna vulgaris*), *Escallonia* spp., all Fabaceae (apart from *Medicago* spp. and *Trifolium* spp.), all Fumariaceae, all Geraniaceae, *Hydrangea* spp., *Hypericum* spp., *Crocosmia* spp., all Lamiaceae (apart from *Lavandula* spp.), *Laurus nobilis*, *Hemerocallis* spp., *Linum* spp., all Malvaceae, *Narthecium ossifragum*, all Oleaceae, all Onagraceae, all Orchidaceae, all Orobanchaceae, *Oxalis* spp., all Papaveraceae, *Mimulus* spp., *Plantago* spp., *Armeria* spp., *Phlox* spp., *Polygala* spp., all Polygonaceae, *Claytonia* spp., all Primulaceae, all Ranunculaceae, all Rosaceae (apart from *Spiraea* spp. and *Prunus lusitanica*), all Rubiaceae, *Choisya* spp., all Scrophulariaceae (apart from *Buddleja* spp., *Veronica pimeleoides*, *Veronica* spp. (subgenus *Pseudoveronica*)*, Veronica speciosa*, all Solanaceae, *Tropaeolum* spp., *Valerianella locusta*, *Viola* spp. |
| Single capitulum | All Asteraceae (except *Solidago canadensis*), *Knautia arvensis* |
| Single branch of capitulas | *Solidago canadensis* |
| Part of panicle | *Spiraea* spp. (apart from *Spiraea douglasii*) |
| Secondary umbel | All Apiaceae |
| Single compound cyme | All Valerianaceae (apart from *Valerianella locusta*) |
| Single corymb | *Cornus* spp., *Sambucus* spp. |
| Single cyme | *Euphorbia* spp. |
| Single panicle | *Buddleja* spp., *Spiraea douglasii* |
| Single raceme | *Calluna vulgaris*, *Medicago* spp., *Prunus lusitanica*, *Trifolium* spp., *Veronica pimeleoides*, *Veronica* spp. (subgenus *Pseudoveronica*), *Veronica speciosa* |
| Single spike | *Callistemon* spp., *Lavandula* spp. |
| Single thyrse | *Ceanothus* spp. |

**Appendix 3. Calculating diversity indices**

**Sørensen similarity index, Proportional Similarity and Horn-Morisita dissimilarity index**

For community comparison analyses visitor taxa identified to species were grouped at the taxonomic level which allowed comparison between sites (94% of individuals were identified to the species level, but for some insects only one gender can be identified to genus or family). Taxa grouped at genus level were *Cyphon* (Coleoptera), *Delia*, *Fannia*, *Helina*, *Oscinella*, *Sarcophaga*, *Sphaerophoria* and *Swammerdamella* (Diptera); taxa grouped at family level were Phoridae, Chironomidae and Dolichopodidae (Diptera).

Three measures were used to assess the similarity in flower-visitor community composition between the 12 sites of each landscape type: (i) Sørensen similarity index (S) to compare the similarity in the species found between sites and (ii) Proportional Similarity (PS; Schoener 1970, Kephart 1983, Horvitz and Schemske 1990) and (iii) Horn-Morisita dissimilarity index (HM) to compare the visitor assemblages between sites. S compares only species’ presence/absence whereas PS and HM take into account the relative proportion of each visitor taxon. HM is included in addition to PS as the index is independent of sample size but at the cost of being insensitive to turnover in rare species. Thus analyses for both PS and HM are retained in the manuscript.

All measures range from one to zero. A higher value of S or PS means that sites are more similar to one another in terms of the species present (S) or flower-visitor assemblages (PS). A higher value of HM means that sites are less similar to one another. The value of PS ranges from one (maximum similarity) to zero (no overlap between assemblages).

Formula for Sørensen:

S = 2a / (2a + b + c) (Shaw 2003)

where a is the total number of species present in both patches being compared, b is the number of species present only in site 1 and c is the number of species present only in site 2.

PS was calculated as follows:

(i) the proportions of flower visits attributable to each visitor taxon were calculated for two sites

(ii) the modulus of the difference in proportions was calculated for each visitor taxon

(iii) PS = 1 - 0.5 (sum of the modulus values over all visitor taxa)

Formula for Horn-Morisita, following Oksanen *et al.* (2013):

$d_{jk}=1- \frac{2\sum_{i} x_{ij}x_{ik}}{(\lambda_{j}+ \lambda_{k})\sum_{i} x_{ij}\sum_{i} x_{ik}}$ , where $\lambda_{j}=\sum_{i} x_{ij}^{2}/{(\sum_{i} x_{ij})}^{2}$

Horvitz, C. C. & Schemske, D. W. 1990 Spatiotemporal variation in insect mutualists of a neotropical herb. *Ecology* **71**:1085-1097. (DOI 10.2307/1937377)

Kephart, S. R. 1983 The partitioning of pollinators among three species of *Asclepias*. *Ecology* **64**:120-133. (DOI 10.2307/1937335)

Oksanen, J. F. Blanchet, G., Kindt, R., Legendre, P., Minchin, P. R., O'Hara, R. B., Simpson, G. L., Solymos, P., Henry, M., Stevens, H. & Wagner, H. 2013 vegan: Community Ecology Package. R package version 2.0-10. http://CRAN.R-project.org/package=vegan

Schoener, T. W. 1970 Nonsynchronous spatial overlap of lizards in patchy habitats. *Ecology* **51**:408-418. (DOI 10.2307/1935376)

Shaw, P. 2003 *Multivariate statistics for the environmental sciences*. New York, NY: Hodder Arnold.

**Appendix 4. Singleton, rare and common taxa across landscape types**

**Table 4.1.** Number of singleton (recorded once in whole study), rare (recorded 2 to 20 times), less common (recorded 21 to 99 times) and common (recorded >100 times in study) visitor taxa found across all 12 urban sites combined, all 12 farmland sites combined and all 12 nature reserve sites combined. Values are also expressed as a percentage of the total number of taxa found for the 12 sites of that landscape type. UR: urban sites, FM: farmland sites, NR: nature reserve sites

|  | **Urban** | | **Farmland** | | **Nature reserve** | |
| --- | --- | --- | --- | --- | --- | --- |
|  | **Number** | **%** | **Number** | **%** | **Number** | **%** |
| Number of species recorded >100 times (common) | 13 | 9.6 | 14 | 5.4 | 14 | 5.3 |
| Number of species recorded 21 to 99 times (less common) | 32 | 23.5 | 38 | 14.7 | 42 | 15.8 |
| Number of species recorded 2 to 20 times (rare) | 64 | 47.0 | 137 | 53.1 | 141 | 53.0 |
| Number of species recorded once (singletons) | 27 | 20.0 | 69 | 26.7 | 69 | 25.9 |
| **Total number species** | **136** |  | **258** |  | **266** |  |

**Table 4.2.** Mean number of local singleton (recorded once at triplet of urban, farmland and nature reserve sites for each city), locally rare (recorded 2 to 10 times), locally less common (recorded 11 to 50 times) and locally common (recorded >50 times) visitor taxa for urban, farmland and nature reserve sites. Values are also expressed as a proportion of the total number of taxa found for the 12 sites of that landscape type. GLMMs were used to compare numbers of taxa in each category between the three landscape types (urban, farmland and nature reserve sites). The effect of landscape type was tested using a log-likelihood ratio test (Zuur *et al.* 2009) comparing models with and without landscape type included. There were 2 degrees of freedom for all analyses. There was no significant difference between landscape types in the numbers of common, less common, rare or singleton species.

|  | **Number of taxa** | | | | | **Proportion of taxa** | | |
| --- | --- | --- | --- | --- | --- | --- | --- | --- |
|  | Mean ± 1SE | | | Effect of landscape type | | Mean ± 1SE | | |
|  | Urban | Farmland | N reserve | χ^2^ | p | Urban | Farmland | N reserve |
| Taxa recorded >50 times (common) | 0.8 ± 0.4 | 0.9 ± 0.4 | 1.0 ± 0.4 | 0.395 | 0.821 | 0.03 ± 0.00 | 0.03 ± 0.01 | 0.03 ± 0.01 |
| Taxa recorded 11 to 50 times (less common) | 7.0 ± 1.6 | 8.3 ± 1.6 | 8.6 ± 1.0 | 0.171 | 0.918 | 0.23 ± 0.01 | 0.18 ± 0.02 | 0.22 ± 0.02 |
| Taxa recorded 2 to 10 times (rare) | 13.9 ± 4.2 | 23.0 ± 4.7 | 21.5 ± 1.8 | 1.198 | 0.549 | 0.47 ± 0.01 | 0.46 ± 0.03 | 0.47 ± 0.03 |
| Taxa recorded once (singletons) | 8.5 ± 4.1 | 14.2 ± 3.0 | 13.2 ± 1.5 | 5.097 | 0.078 | 0.27 ± 0.02 | 0.33 ± 0.03 | 0.29 ± 0.03 |

**Appendix 5. Rare and scarce UK species recorded**

**Table 5.1 Rare and scarce UK species recorded in the study**

| **Order** | **Species** | **Status** | **Sites recorded at** |
| --- | --- | --- | --- |
| Hymenoptera | *Bombus humilis* | BAP Priority Species | Cardiff urban, farm, nature reserve |
| Lepidoptera | *Boloria selene* | BAP Priority Species | Glasgow nature reserve |
| Lepidoptera | *Coenonympha pamphilus* | BAP Priority Species | Dundee nature reserve, Swindon nature reserve |
| Diptera: Syrphidae | *Cheilosia cynocephala* | Nationally scarce^1^ | Cardiff farm |
| Diptera: Syrphidae | *Cheilosia velutina* | Nationally scarce^1^ | London urban |
| Diptera: Syrphidae | *Neoascia interrupta* | Nationally scarce^1^ | Hull nature reserve |
| Diptera: Syrphidae | *Pelecocera tricincta* | Nationally scarce^1^ | Reading nature reserve |
| Hymenoptera | *Anthophora quadrimaculata* | Notable species Nb^2^ | London urban |
| Hymenoptera | *Bombus rupestris* | Notable species Nb^2^ | London farm, Hull nature reserve |
| Hymenoptera | *Lasioglossum malachura* | Notable species Nb^2^ | Bristol farm |
| Hymenoptera | *Lasioglossum pauxillum* | Notable species Na^2^ | Reading farm, Southampton farm, London urban |

Na Estimated to occur within the range of 16-30 10km squares

Nb Estimated to occur within the range of 31-100 10km squares

**References**

1. Ball, S. & Morris, R. 2013 Britain's Hoverflies: An Introduction to the Hoverflies of Britain. Princeton University Press, New Jersey/Oxfordshire 296pp

2. Falk 1991 *A Review of the Scarce and Threatened Bees, Wasp and Ants of Great Britain.* Research and Survey in Nature Conservation Report no. 35. Available to download from: http://www.bwars.com/index.php?q=content/uk-guides-national-regional-and-county-guides-aculeates

**Appendix 6. NMDS plot of the 36 flower-visitor communities**

**Figure 6.1.** Non-metric multidimensional scaling plot summarising variation among the 36 flower-visitor communities. Urban sites = white squares, farmland sites = black triangles, nature reserves = white circles. Stress: 0.196

**Appendix 7. Plant-pollinator network metrics across landscape types**

**Table 7.1** Results of GLMMs testing for differences in evenness, visitor and plant generality and plant species richness between the three landscape types (urban, farmland and nature reserve sites). The effect of landscape type was tested using a log-likelihood ratio test (Zuur *et al.* 2009) comparing models with and without landscape type included. There were 2 degrees of freedom for all analyses. Significant *post hoc* Tukey tests used to test for differences between landscape pairs are shown, near-significant p-values are given in brackets and all other pairwise comparisons were not significant. Means and standard errors are calculated from the raw data. FM: farmland sites, NR: nature reserve sites, UR: urban sites.

| **Network metric** | **Mean ± 1SE** | | | **Effect of landscape type** | | **Tukey *post hoc* tests** |
| --- | --- | --- | --- | --- | --- | --- |
|  | **Urban** | **Farmland** | **Nature reserve** | **χ^2^** | **p** |  |
| Plant generality | 3.731 ± 0.375 | 8.118 ± 1.095 | 6.915 ± 0.949 | 20.261 | <**0.0001** | FM>UR p<**0.001**  NR>UR p<**0.001** |
| Visitor generality | 3.96 ± 0.40 | 2.18 ± 0.21 | 2.21 ± 0.20 | 12.498 | **0.0019** | FM<UR p= **0.025**  NR<UR p= **0.037** |
| Species-level specialisation: plants (d’) ^a^ | 0.52 ± 0.04 | 0.60 ± 0.04 | 0.54 ± 0.02 | 3.599 | 0.1654 | - |
| Species-level specialisation: visitors (d’) ^a^ | 0.48 ± 0.03 | 0.30 ± 0.02 | 0.33 ± 0.03 | 24.143 | <**0.0001** | FM<UR p<**0.001**  NR<UR p< **0.001** |
| Network-level specialisation (H2’) ^a^ | 0.47 ± 0.03 | 0.58 ± 0.04 | 0.50 ± 0.03 | 8.801 | **0.012** | NR-FM ns (p=0.062)  FM>UR p= **0.001** |
| Floral richness: overall | 41.25 ± 2.91 | 22.00 ± 2.26 | 23.67 ± 4.90 | 10.305 | **0.006** | FM<UR p= **0.001**  NR<UR ns (p=0.053) |
| Floral richness: native | 22.92 ± 1.90 | 20.50 ± 2.25 | 23.25 ± 4.78 | 0.681 | 0.712 | - |
| Floral richness: non-native | 18.33 ± 2.40 | 1.50 ± 0.34 | 0.42 ± 0.19 | 53.737 | <**0.0001** | FM<UR p<**0.001**  NR<UR p< **0.001** |
| Number of visits: native plants | 78.00 ± 13.93 | 195.58 ± 43.61 | 257.42 ± 65.56 | 13.780 | **0.001** | FM>UR p=**0.008**  NR>UR p< **0.001** |
| Number of visits: non-native plants | 56.83 ± 11.37 | 27.00 ± 17.24 | 2.83 ± 1.94 | 22.801 | <**0.0001** | FM<UR p<**0.001**  NR<UR p<**0.001** |

^a^ Note it was not possible to calculate these values for the Sheffield nature reserve site as the network was too small (one plant species only), thus analyses for these response variables across landscape types excludes the triplet of sites for Sheffield.
