# Supplementary material for: Where is the UK's pollinator biodiversity? The importance of urban areas for flower-visiting insects
Source: Proc Biol Sci. 2015 Mar 22;282(1803):20142849. doi: 10.1098/rspb.2014.2849 (PMC4345454; doi:10.1098/rspb.2014.2849)
Supplement: Datasets [file rspb20142849supp2.pdf]

# Electronic Supplementary Material 2: Datasets used in analyses

**Table 1 Flower visitor abundance recorded across all whole sampling season per site**

| Site | All taxa | Coleoptera | Diptera | Syrphidae | Hymenoptera | Lepidoptera | Bees | Honeybees | Bumblebees | Solitary bees |
|---|---|---|---|---|---|---|---|---|---|---|
| Bristol farmland | 302 | 17 | 203 | 55 | 77 | 5 | 73 | 33 | 21 | 19 |
| Bristol nature reserve | 152 | 14 | 69 | 26 | 50 | 19 | 40 | 10 | 19 | 11 |
| Bristol urban | 211 | 2 | 48 | 30 | 161 | 0 | 154 | 76 | 52 | 26 |
| Cardiff farmland | 521 | 21 | 440 | 124 | 54 | 6 | 48 | 17 | 27 | 4 |
| Cardiff nature reserve | 281 | 19 | 188 | 68 | 72 | 2 | 71 | 35 | 29 | 7 |
| Cardiff urban | 189 | 4 | 69 | 15 | 116 | 0 | 29 | 1 | 21 | 7 |
| Dundee farmland | 349 | 12 | 278 | 144 | 46 | 13 | 40 | 0 | 38 | 2 |
| Dundee nature reserve | 375 | 15 | 333 | 116 | 23 | 4 | 17 | 0 | 15 | 2 |
| Dundee urban | 213 | 6 | 153 | 92 | 40 | 14 | 33 | 2 | 30 | 1 |
| Edinburgh farmland | 112 | 2 | 104 | 26 | 6 | 0 | 3 | 0 | 3 | 0 |
| Edinburgh nature reserve | 304 | 29 | 256 | 111 | 16 | 3 | 12 | 0 | 12 | 0 |
| Edinburgh urban | 99 | 2 | 52 | 43 | 45 | 0 | 45 | 2 | 40 | 3 |
| Glasgow farmland | 409 | 12 | 376 | 96 | 17 | 4 | 14 | 0 | 14 | 0 |
| Glasgow nature reserve | 381 | 37 | 296 | 70 | 30 | 18 | 25 | 0 | 25 | 0 |
| Glasgow urban | 23 | 0 | 19 | 10 | 4 | 0 | 1 | 0 | 1 | 0 |
| Hull farmland | 97 | 10 | 58 | 49 | 28 | 1 | 0 | 5 | 20 | 0 |
| Hull nature reserve | 794 | 25 | 569 | 451 | 199 | 1 | 192 | 17 | 175 | 0 |
| Hull urban | 174 | 10 | 136 | 113 | 28 | 0 | 28 | 1 | 26 | 1 |
| Leeds farmland | 121 | 2 | 47 | 14 | 71 | 1 | 66 | 19 | 47 | 0 |
| Leeds nature reserve | 54 | 15 | 24 | 13 | 14 | 1 | 12 | 3 | 9 | 0 |
| Leeds urban | 152 | 0 | 64 | 61 | 88 | 0 | 86 | 17 | 66 | 3 |
| London farmland | 219 | 15 | 42 | 23 | 160 | 2 | 156 | 56 | 95 | 5 |
| London nature reserve | 29 | 0 | 24 | 23 | 5 | 0 | 5 | 0 | 5 | 0 |
| London urban | 182 | 37 | 48 | 28 | 94 | 3 | 85 | 24 | 46 | 15 |

| | | | | | | | | | | |
|---|---|---|---|---|---|---|---|---|---|---|
| Reading farmland | 212 | 27 | 146 | 71 | 37 | 2 | 31 | 9 | 5 | 17 |
| Reading nature reserve | 51 | 1 | 25 | 23 | 25 | 0 | 22 | 11 | 11 | 0 |
| Reading urban | 90 | 3 | 45 | 31 | 42 | 0 | 41 | 8 | 25 | 8 |
| Sheffield farmland | 54 | 3 | 34 | 18 | 17 | 0 | 15 | 7 | 7 | 1 |
| Sheffield nature reserve | 223 | 0 | 159 | 158 | 64 | 0 | 62 | 44 | 18 | 0 |
| Sheffield urban | 96 | 2 | 61 | 60 | 32 | 1 | 32 | 5 | 24 | 3 |
| Southampton farmland | 251 | 7 | 149 | 59 | 94 | 1 | 92 | 53 | 30 | 9 |
| Southampton nature reserve | 14 | 0 | 9 | 7 | 5 | 0 | 5 | 2 | 2 | 1 |
| Southampton urban | 87 | 1 | 14 | 12 | 72 | 0 | 71 | 34 | 34 | 3 |
| Swindon farmland | 24 | 1 | 17 | 10 | 6 | 0 | 3 | 3 | 0 | 0 |
| Swindon nature reserve | 465 | 19 | 361 | 63 | 47 | 38 | 32 | 4 | 25 | 3 |
| Swindon urban | 102 | 3 | 43 | 26 | 53 | 3 | 53 | 4 | 48 | 1 |

**Table 2 Flower visitor abundance recorded per sampling month per site**

| Site | Month | All taxa | Coleoptera | Diptera | Syrphidae | Hymenoptera | Lepidoptera | Bees | Honeybees | Bumblebees | Solitary bees |
|---|---|---|---|---|---|---|---|---|---|---|---|
| Bristol farmland | June | 106 | 5 | 74 | 23 | 4 | 18 | 23 | 14 | 6 | 3 |
| Bristol farmland | July | 109 | 12 | 57 | 39 | 1 | 19 | 39 | 19 | 11 | 9 |
| Bristol farmland | August | 47 | 0 | 36 | 11 | 0 | 5 | 8 | 0 | 3 | 5 |
| Bristol farmland | September | 40 | 0 | 36 | 4 | 0 | 13 | 3 | 0 | 1 | 2 |
| Bristol nature reserve | June | 41 | 9 | 14 | 9 | 9 | 8 | 7 | 4 | 3 | 0 |
| Bristol nature reserve | July | 42 | 5 | 17 | 11 | 9 | 11 | 11 | 3 | 8 | 0 |
| Bristol nature reserve | August | 65 | 0 | 36 | 28 | 1 | 6 | 20 | 3 | 8 | 9 |
| Bristol nature reserve | September | 4 | 0 | 2 | 2 | 0 | 1 | 2 | 0 | 0 | 2 |
| Bristol urban | June | 30 | 2 | 14 | 14 | 0 | 8 | 13 | 3 | 2 | 8 |
| Bristol urban | July | 46 | 0 | 11 | 35 | 0 | 6 | 31 | 8 | 10 | 13 |
| Bristol urban | August | 80 | 0 | 7 | 73 | 0 | 4 | 72 | 36 | 31 | 5 |
| Bristol urban | September | 55 | 0 | 16 | 39 | 0 | 12 | 38 | 29 | 9 | 0 |
| Cardiff farmland | June | 161 | 11 | 128 | 17 | 5 | 38 | 15 | 5 | 9 | 1 |
| Cardiff farmland | July | 72 | 7 | 53 | 12 | 0 | 15 | 11 | 4 | 5 | 2 |
| Cardiff farmland | August | 209 | 2 | 186 | 21 | 0 | 45 | 18 | 8 | 10 | 0 |
| Cardiff farmland | September | 79 | 1 | 73 | 4 | 1 | 26 | 4 | 0 | 3 | 1 |
| Cardiff nature reserve | June | 45 | 2 | 26 | 17 | 0 | 11 | 17 | 7 | 8 | 2 |
| Cardiff nature reserve | July | 97 | 15 | 56 | 25 | 1 | 13 | 25 | 17 | 4 | 4 |
| Cardiff nature reserve | August | 79 | 1 | 55 | 22 | 1 | 10 | 21 | 11 | 10 | 0 |
| Cardiff nature reserve | September | 60 | 1 | 51 | 8 | 0 | 34 | 8 | 0 | 7 | 1 |
| Cardiff urban | June | 25 | 4 | 9 | 12 | 0 | 4 | 11 | 0 | 7 | 4 |
| Cardiff urban | July | 12 | 0 | 5 | 7 | 0 | 4 | 6 | 0 | 5 | 1 |
| Cardiff urban | August | 135 | 0 | 43 | 92 | 0 | 4 | 8 | 1 | 6 | 1 |
| Cardiff urban | September | 17 | 0 | 12 | 5 | 0 | 3 | 4 | 0 | 3 | 1 |
| Dundee farmland | June | 140 | 11 | 73 | 43 | 13 | 23 | 38 | 0 | 36 | 2 |
| Dundee farmland | July | 69 | 1 | 67 | 1 | 0 | 6 | 1 | 0 | 1 | 0 |
| Dundee farmland | August | 89 | 0 | 88 | 1 | 0 | 72 | 1 | 0 | 1 | 0 |

| | | | | | | | | | | | |
|---|---|---|---|---|---|---|---|---|---|---|---|
| Dundee farmland | September | 51 | 0 | 50 | 1 | 0 | 43 | 0 | 0 | 0 | 0 |
| Dundee nature reserve | June | 90 | 4 | 73 | 12 | 1 | 7 | 10 | 0 | 8 | 2 |
| Dundee nature reserve | July | 116 | 6 | 102 | 7 | 1 | 9 | 5 | 0 | 5 | 0 |
| Dundee nature reserve | August | 144 | 1 | 137 | 4 | 2 | 82 | 2 | 0 | 2 | 0 |
| Dundee nature reserve | September | 25 | 4 | 21 | 0 | 0 | 18 | 0 | 0 | 0 | 0 |
| Dundee urban | June | 52 | 6 | 15 | 17 | 14 | 0 | 13 | 0 | 13 | 0 |
| Dundee urban | July | 14 | 0 | 9 | 5 | 0 | 0 | 4 | 0 | 4 | 0 |
| Dundee urban | August | 103 | 0 | 92 | 11 | 0 | 60 | 9 | 1 | 8 | 0 |
| Dundee urban | September | 44 | 0 | 37 | 7 | 0 | 32 | 7 | 1 | 5 | 1 |
| Edinburgh farmland | June | 44 | 1 | 40 | 3 | 0 | 2 | 1 | 0 | 1 | 0 |
| Edinburgh farmland | July | 44 | 1 | 42 | 1 | 0 | 2 | 0 | 0 | 0 | 0 |
| Edinburgh farmland | August | 24 | 0 | 22 | 2 | 0 | 22 | 2 | 0 | 2 | 0 |
| Edinburgh farmland | September | 0 | 0 | 0 | 0 | 0 | 0 | 0 | 0 | 0 | 0 |
| Edinburgh nature reserve | June | 164 | 26 | 123 | 12 | 3 | 5 | 10 | 0 | 10 | 0 |
| Edinburgh nature reserve | July | 10 | 0 | 7 | 3 | 0 | 1 | 1 | 0 | 1 | 0 |
| Edinburgh nature reserve | August | 127 | 1 | 125 | 1 | 0 | 104 | 1 | 0 | 1 | 0 |
| Edinburgh nature reserve | September | 3 | 2 | 1 | 0 | 0 | 1 | 0 | 0 | 0 | 0 |
| Edinburgh urban | June | 10 | 0 | 3 | 7 | 0 | 0 | 7 | 0 | 7 | 0 |
| Edinburgh urban | July | 20 | 2 | 4 | 14 | 0 | 1 | 13 | 2 | 11 | 1 |
| Edinburgh urban | August | 63 | 0 | 42 | 21 | 0 | 39 | 20 | 0 | 20 | 1 |
| Edinburgh urban | September | 6 | 0 | 3 | 3 | 0 | 3 | 2 | 0 | 2 | 1 |
| Glasgow farmland | June | 209 | 10 | 195 | 1 | 3 | 18 | 1 | 0 | 1 | 0 |
| Glasgow farmland | July | 95 | 2 | 79 | 13 | 1 | 24 | 12 | 0 | 12 | 0 |
| Glasgow farmland | August | 52 | 0 | 50 | 2 | 0 | 17 | 1 | 0 | 1 | 0 |
| Glasgow farmland | September | 53 | 0 | 52 | 1 | 0 | 37 | 0 | 0 | 0 | 0 |
| Glasgow nature reserve | June | 133 | 21 | 96 | 5 | 11 | 12 | 3 | 0 | 3 | 0 |
| Glasgow nature reserve | July | 104 | 9 | 75 | 13 | 7 | 9 | 12 | 0 | 12 | 0 |

| | | | | | | | | | | | |
|---|---|---|---|---|---|---|---|---|---|---|---|
| Glasgow nature reserve | August | 78 | 3 | 67 | 8 | 0 | 15 | 8 | 0 | 8 | 0 |
| Glasgow nature reserve | September | 66 | 4 | 58 | 4 | 0 | 34 | 2 | 0 | 2 | 0 |
| Glasgow urban | June | 3 | 0 | 2 | 1 | 0 | 1 | 1 | 0 | 1 | 0 |
| Glasgow urban | July | 3 | 0 | 3 | 0 | 0 | 0 | 0 | 0 | 0 | 0 |
| Glasgow urban | August | 10 | 0 | 7 | 3 | 0 | 2 | 0 | 0 | 0 | 0 |
| Glasgow urban | September | 7 | 0 | 7 | 0 | 0 | 7 | 0 | 0 | 0 | 0 |
| Hull farmland | June | 34 | 5 | 9 | 20 | 0 | 4 | 0 | 2 | 16 | 0 |
| Hull farmland | July | 14 | 4 | 3 | 6 | 1 | 1 | 0 | 3 | 3 | 0 |
| Hull farmland | August | 47 | 0 | 45 | 2 | 0 | 44 | 0 | 0 | 1 | 0 |
| Hull farmland | September | 2 | 1 | 1 | 0 | 0 | 0 | 0 | 0 | 0 | 0 |
| Hull nature reserve | June | 87 | 0 | 46 | 41 | 0 | 23 | 39 | 0 | 39 | 0 |
| Hull nature reserve | July | 110 | 11 | 14 | 85 | 0 | 8 | 84 | 3 | 81 | 0 |
| Hull nature reserve | August | 503 | 11 | 432 | 59 | 1 | 408 | 55 | 14 | 41 | 0 |
| Hull nature reserve | September | 94 | 3 | 77 | 14 | 0 | 12 | 14 | 0 | 14 | 0 |
| Hull urban | June | 33 | 10 | 13 | 10 | 0 | 0 | 10 | 0 | 10 | 0 |
| Hull urban | July | 17 | 0 | 3 | 14 | 0 | 2 | 14 | 0 | 14 | 0 |
| Hull urban | August | 112 | 0 | 109 | 3 | 0 | 105 | 3 | 1 | 1 | 1 |
| Hull urban | September | 12 | 0 | 11 | 1 | 0 | 6 | 1 | 0 | 1 | 0 |
| Leeds farmland | June | 44 | 2 | 1 | 40 | 1 | 1 | 40 | 12 | 28 | 0 |
| Leeds farmland | July | 18 | 0 | 10 | 8 | 0 | 1 | 8 | 0 | 8 | 0 |
| Leeds farmland | August | 33 | 0 | 15 | 18 | 0 | 8 | 18 | 7 | 11 | 0 |
| Leeds farmland | September | 26 | 0 | 21 | 5 | 0 | 4 | 0 | 0 | 0 | 0 |
| Leeds nature reserve | June | 24 | 11 | 5 | 8 | 0 | 2 | 0 | 3 | 5 | 0 |
| Leeds nature reserve | July | 17 | 4 | 9 | 3 | 1 | 4 | 0 | 0 | 2 | 0 |
| Leeds nature reserve | August | 7 | 0 | 6 | 1 | 0 | 5 | 0 | 0 | 1 | 0 |
| Leeds nature reserve | September | 6 | 0 | 4 | 2 | 0 | 2 | 0 | 0 | 1 | 0 |
| Leeds urban | June | 76 | 0 | 4 | 72 | 0 | 2 | 71 | 13 | 56 | 2 |
| Leeds urban | July | 7 | 0 | 4 | 3 | 0 | 3 | 2 | 1 | 1 | 0 |

| | | | | | | | | | | | |
|---|---|---|---|---|---|---|---|---|---|---|---|
| Leeds urban | August | 40 | 0 | 29 | 11 | 0 | 29 | 11 | 1 | 9 | 1 |
| Leeds urban | September | 29 | 0 | 27 | 2 | 0 | 27 | 2 | 2 | 0 | 0 |
| London farmland | June | 98 | 2 | 2 | 94 | 0 | 2 | 92 | 17 | 75 | 0 |
| London farmland | July | 28 | 9 | 3 | 14 | 2 | 2 | 13 | 11 | 2 | 0 |
| London farmland | August | 81 | 4 | 33 | 44 | 0 | 17 | 44 | 28 | 11 | 5 |
| London farmland | September | 12 | 0 | 4 | 8 | 0 | 2 | 7 | 0 | 7 | 0 |
| London nature reserve | June | 0 | 0 | 0 | 0 | 0 | 0 | 0 | 0 | 0 | 0 |
| London nature reserve | July | 2 | 0 | 1 | 1 | 0 | 1 | 0 | 0 | 1 | 0 |
| London nature reserve | August | 23 | 0 | 20 | 3 | 0 | 19 | 0 | 0 | 3 | 0 |
| London nature reserve | September | 4 | 0 | 3 | 1 | 0 | 3 | 0 | 0 | 1 | 0 |
| London urban | June | 40 | 12 | 6 | 21 | 1 | 2 | 21 | 6 | 15 | 0 |
| London urban | July | 75 | 20 | 25 | 28 | 2 | 14 | 21 | 3 | 10 | 8 |
| London urban | August | 41 | 5 | 12 | 24 | 0 | 11 | 23 | 12 | 7 | 4 |
| London urban | September | 26 | 0 | 5 | 21 | 0 | 1 | 20 | 3 | 14 | 3 |
| Reading farmland | June | 77 | 20 | 44 | 12 | 1 | 16 | 9 | 5 | 2 | 2 |
| Reading farmland | July | 133 | 7 | 100 | 25 | 1 | 55 | 22 | 4 | 3 | 15 |
| Reading farmland | August | 2 | 0 | 2 | 0 | 0 | 0 | 0 | 0 | 0 | 0 |
| Reading farmland | September | 0 | 0 | 0 | 0 | 0 | 0 | 0 | 0 | 0 | 0 |
| Reading nature reserve | June | 2 | 1 | 0 | 1 | 0 | 0 | 1 | 0 | 1 | 0 |
| Reading nature reserve | July | 22 | 0 | 18 | 4 | 0 | 18 | 1 | 0 | 1 | 0 |
| Reading nature reserve | August | 12 | 0 | 3 | 9 | 0 | 1 | 9 | 6 | 3 | 0 |
| Reading nature reserve | September | 15 | 0 | 4 | 11 | 0 | 4 | 11 | 5 | 6 | 0 |
| Reading urban | June | 23 | 2 | 7 | 14 | 0 | 2 | 14 | 0 | 14 | 0 |
| Reading urban | July | 51 | 1 | 29 | 21 | 0 | 24 | 20 | 6 | 7 | 7 |
| Reading urban | August | 8 | 0 | 5 | 3 | 0 | 3 | 3 | 1 | 2 | 0 |
| Reading urban | September | 8 | 0 | 4 | 4 | 0 | 2 | 4 | 1 | 2 | 1 |
| Sheffield farmland | June | 11 | 2 | 7 | 2 | 0 | 0 | 2 | 0 | 1 | 1 |
| Sheffield farmland | July | 10 | 1 | 4 | 5 | 0 | 2 | 3 | 0 | 3 | 0 |

| | | | | | | | | | | | |
|---|---|---|---|---|---|---|---|---|---|---|---|
| Sheffield farmland | August | 19 | 0 | 11 | 8 | 0 | 11 | 8 | 7 | 1 | 0 |
| Sheffield farmland | September | 14 | 0 | 12 | 2 | 0 | 5 | 2 | 0 | 2 | 0 |
| Sheffield nature reserve | June | 0 | 0 | 0 | 0 | 0 | 0 | 0 | 0 | 0 | 0 |
| Sheffield nature reserve | July | 0 | 0 | 0 | 0 | 0 | 0 | 0 | 0 | 0 | 0 |
| Sheffield nature reserve | August | 221 | 0 | 159 | 62 | 0 | 158 | 60 | 44 | 16 | 0 |
| Sheffield nature reserve | September | 2 | 0 | 0 | 2 | 0 | 0 | 2 | 0 | 2 | 0 |
| Sheffield urban | June | 18 | 0 | 2 | 16 | 0 | 2 | 16 | 2 | 13 | 1 |
| Sheffield urban | July | 9 | 0 | 2 | 7 | 0 | 1 | 7 | 3 | 2 | 2 |
| Sheffield urban | August | 52 | 2 | 41 | 8 | 1 | 41 | 8 | 0 | 8 | 0 |
| Sheffield urban | September | 17 | 0 | 16 | 1 | 0 | 16 | 1 | 0 | 1 | 0 |
| Southampton farmland | June | 1 | 0 | 1 | 0 | 0 | 0 | 0 | 0 | 0 | 0 |
| Southampton farmland | July | 83 | 6 | 54 | 23 | 0 | 36 | 23 | 10 | 7 | 6 |
| Southampton farmland | August | 101 | 1 | 47 | 52 | 1 | 3 | 41 | 36 | 13 | 2 |
| Southampton farmland | September | 66 | 0 | 47 | 19 | 0 | 20 | 18 | 7 | 10 | 1 |
| Southampton nature reserve | June | 1 | 0 | 1 | 0 | 0 | 1 | 0 | 0 | 0 | 0 |
| Southampton nature reserve | July | 6 | 0 | 4 | 2 | 0 | 3 | 2 | 0 | 1 | 1 |
| Southampton nature reserve | August | 1 | 0 | 0 | 1 | 0 | 0 | 1 | 1 | 0 | 0 |
| Southampton nature reserve | September | 6 | 0 | 4 | 2 | 0 | 3 | 2 | 1 | 1 | 0 |
| Southampton urban | June | 22 | 0 | 1 | 21 | 0 | 1 | 21 | 6 | 15 | 0 |
| Southampton urban | July | 31 | 1 | 2 | 28 | 0 | 2 | 27 | 14 | 11 | 2 |
| Southampton urban | August | 25 | 0 | 9 | 16 | 0 | 8 | 16 | 14 | 2 | 0 |
| Southampton urban | September | 9 | 0 | 2 | 7 | 0 | 1 | 7 | 0 | 6 | 1 |
| Swindon farmland | June | 2 | 0 | 0 | 2 | 0 | 0 | 0 | 0 | 0 | 0 |
| Swindon farmland | July | 0 | 0 | 0 | 0 | 0 | 0 | 0 | 0 | 0 | 0 |
| Swindon farmland | August | 10 | 1 | 6 | 3 | 0 | 4 | 3 | 3 | 0 | 0 |
| Swindon farmland | September | 12 | 0 | 11 | 1 | 0 | 6 | 0 | 0 | 0 | 0 |

| | | | | | | | | | | | |
|---|---|---|---|---|---|---|---|---|---|---|---|
| Swindon nature reserve | June | 17 | 6 | 10 | 1 | 0 | 0 | 0 | 0 | 0 | 0 |
| Swindon nature reserve | July | 125 | 1 | 104 | 11 | 9 | 4 | 11 | 0 | 10 | 1 |
| Swindon nature reserve | August | 122 | 9 | 78 | 12 | 23 | 47 | 10 | 2 | 6 | 2 |
| Swindon nature reserve | September | 201 | 3 | 169 | 23 | 6 | 12 | 11 | 2 | 9 | 0 |
| Swindon urban | June | 8 | 2 | 2 | 3 | 1 | 1 | 3 | 0 | 2 | 1 |
| Swindon urban | July | 25 | 1 | 13 | 11 | 0 | 6 | 11 | 2 | 9 | 0 |
| Swindon urban | August | 42 | 0 | 18 | 22 | 2 | 12 | 22 | 2 | 20 | 0 |
| Swindon urban | September | 27 | 0 | 10 | 17 | 0 | 7 | 17 | 0 | 17 | 0 |

**Table 3 Flower visitor richness recorded across whole sampling season per site**

| Site | *All taxa* | *Coleoptera* | *Diptera* | *Syrphidae* | *Hymenoptera* | *Lepidoptera* | *Bees* | *Bumblebees* | *Solitary bees* |
|---|---|---|---|---|---|---|---|---|---|
| Bristol farmland | 63 | 4 | 41 | 15 | 3 | 16 | 12 | 5 | 6 |
| Bristol nature reserve | 51 | 8 | 24 | 15 | 5 | 10 | 9 | 5 | 3 |
| Bristol urban | 42 | 2 | 25 | 15 | 0 | 13 | 11 | 4 | 6 |
| Cardiff farmland | 83 | 9 | 57 | 13 | 4 | 22 | 9 | 5 | 3 |
| Cardiff nature reserve | 56 | 4 | 39 | 11 | 2 | 20 | 10 | 6 | 3 |
| Cardiff urban | 42 | 3 | 24 | 15 | 0 | 8 | 10 | 5 | 4 |
| Dundee farmland | 78 | 7 | 59 | 9 | 3 | 15 | 7 | 5 | 2 |
| Dundee nature reserve | 87 | 7 | 65 | 11 | 4 | 18 | 7 | 5 | 2 |
| Dundee urban | 48 | 3 | 31 | 12 | 2 | 12 | 7 | 5 | 1 |
| Edinburgh farmland | 29 | 2 | 22 | 5 | 0 | 4 | 2 | 2 | 0 |
| Edinburgh nature reserve | 61 | 6 | 44 | 8 | 3 | 15 | 5 | 5 | 0 |
| Edinburgh urban | 24 | 1 | 12 | 11 | 0 | 6 | 11 | 7 | 3 |
| Glasgow farmland | 76 | 3 | 63 | 6 | 4 | 23 | 4 | 4 | 0 |
| Glasgow nature reserve | 82 | 12 | 57 | 9 | 4 | 22 | 4 | 4 | 0 |
| Glasgow urban | 17 | 0 | 14 | 3 | 0 | 5 | 1 | 1 | 0 |
| Hull farmland | 30 | 5 | 14 | 10 | 1 | 9 | 7 | 6 | 0 |
| Hull nature reserve | 79 | 6 | 58 | 14 | 1 | 26 | 10 | 9 | 0 |
| Hull urban | 27 | 1 | 20 | 6 | 0 | 8 | 6 | 4 | 1 |
| Leeds farmland | 24 | 2 | 15 | 6 | 1 | 6 | 5 | 4 | 0 |
| Leeds nature reserve | 25 | 5 | 12 | 7 | 1 | 6 | 6 | 5 | 0 |
| Leeds urban | 28 | 0 | 15 | 13 | 0 | 12 | 11 | 8 | 2 |
| London farmland | 36 | 6 | 13 | 15 | 2 | 8 | 11 | 6 | 4 |
| London nature reserve | 7 | 0 | 5 | 2 | 0 | 4 | 2 | 2 | 0 |
| London urban | 53 | 6 | 23 | 22 | 2 | 10 | 18 | 5 | 12 |
| Reading farmland | 66 | 6 | 41 | 17 | 2 | 15 | 13 | 4 | 8 |
| Reading nature reserve | 17 | 1 | 9 | 7 | 0 | 7 | 7 | 5 | 1 |

| | | | | | | | | | |
|---|---|---|---|---|---|---|---|---|---|
| Reading urban | 36 | 3 | 19 | 14 | 0 | 10 | 13 | 5 | 7 |
| Sheffield farmland | 26 | 3 | 14 | 9 | 0 | 6 | 7 | 5 | 1 |
| Sheffield nature reserve | 15 | 0 | 10 | 5 | 0 | 9 | 3 | 2 | 0 |
| Sheffield urban | 20 | 2 | 8 | 9 | 1 | 7 | 9 | 6 | 2 |
| Southampton farmland | 53 | 2 | 39 | 11 | 1 | 17 | 9 | 2 | 6 |
| Southampton nature reserve | 8 | 0 | 5 | 3 | 0 | 3 | 3 | 1 | 1 |
| Southampton urban | 15 | 1 | 5 | 9 | 0 | 4 | 8 | 5 | 2 |
| Swindon farmland | 15 | 1 | 11 | 3 | 0 | 5 | 1 | 0 | 0 |
| Swindon nature reserve | 67 | 7 | 36 | 16 | 8 | 9 | 9 | 6 | 2 |
| Swindon urban | 28 | 2 | 17 | 7 | 2 | 9 | 7 | 5 | 1 |

**Table 4 Flower visitor richness and floral unit abundance recorded per sampling month per site**

| Site | Month | All taxa | Coleoptera | Diptera | Syrphidae | Hymenoptera | Lepidoptera | Bees | Bumblebees | Solitary bees | Floral unit abundance |
|---|---|---|---|---|---|---|---|---|---|---|---|
| Bristol farmland | June | 35 | 3 | 24 | 6 | 2 | 7 | 6 | 3 | 2 | 395 |
| Bristol farmland | July | 28 | 3 | 15 | 9 | 1 | 8 | 9 | 4 | 4 | 284 |
| Bristol farmland | August | 19 | 0 | 13 | 6 | 0 | 5 | 4 | 1 | 3 | 23 |
| Bristol farmland | September | 16 | 0 | 13 | 3 | 0 | 6 | 2 | 1 | 1 | 13 |
| Bristol nature reserve | June | 21 | 6 | 7 | 5 | 3 | 3 | 3 | 2 | 0 | 102 |
| Bristol nature reserve | July | 20 | 2 | 9 | 5 | 4 | 6 | 5 | 4 | 0 | 182 |
| Bristol nature reserve | August | 27 | 0 | 16 | 10 | 1 | 6 | 6 | 2 | 3 | 18 |
| Bristol nature reserve | September | 3 | 0 | 2 | 1 | 0 | 1 | 1 | 0 | 1 | 5 |
| Bristol urban | June | 18 | 2 | 8 | 8 | 0 | 4 | 7 | 2 | 4 | 574 |
| Bristol urban | July | 20 | 0 | 10 | 10 | 0 | 5 | 7 | 2 | 4 | 303 |
| Bristol urban | August | 15 | 0 | 6 | 9 | 0 | 3 | 8 | 4 | 3 | 306 |
| Bristol urban | September | 14 | 0 | 10 | 4 | 0 | 7 | 3 | 2 | 0 | 131 |
| Cardiff farmland | June | 49 | 7 | 33 | 6 | 3 | 13 | 5 | 3 | 1 | 721 |
| Cardiff farmland | July | 25 | 2 | 16 | 7 | 0 | 6 | 6 | 3 | 2 | 377 |
| Cardiff farmland | August | 39 | 2 | 30 | 7 | 0 | 14 | 5 | 4 | 0 | 902 |
| Cardiff farmland | September | 18 | 1 | 14 | 2 | 1 | 9 | 2 | 1 | 1 | 21 |
| Cardiff nature reserve | June | 27 | 1 | 19 | 7 | 0 | 8 | 7 | 4 | 2 | 377 |
| Cardiff nature reserve | July | 28 | 2 | 19 | 6 | 1 | 10 | 6 | 4 | 1 | 519 |
| Cardiff nature reserve | August | 19 | 1 | 13 | 4 | 1 | 6 | 3 | 2 | 0 | 581 |
| Cardiff nature reserve | September | 19 | 1 | 15 | 3 | 0 | 9 | 3 | 2 | 1 | 123 |
| Cardiff urban | June | 15 | 3 | 5 | 7 | 0 | 3 | 6 | 3 | 3 | 169 |
| Cardiff urban | July | 9 | 0 | 4 | 5 | 0 | 3 | 4 | 3 | 1 | 135 |
| Cardiff urban | August | 26 | 0 | 17 | 9 | 0 | 4 | 6 | 4 | 1 | 102 |
| Cardiff urban | September | 10 | 0 | 7 | 3 | 0 | 2 | 2 | 1 | 1 | 67 |
| Dundee farmland | June | 44 | 6 | 26 | 9 | 3 | 5 | 7 | 5 | 2 | 1633 |
| Dundee farmland | July | 29 | 1 | 27 | 1 | 0 | 6 | 1 | 1 | 0 | 510 |

| | | | | | | | | | | | |
|---|---|---|---|---|---|---|---|---|---|---|---|
| Dundee farmland | August | 15 | 0 | 14 | 1 | 0 | 1 | 1 | 1 | 0 | 313 |
| Dundee farmland | September | 14 | 0 | 13 | 1 | 0 | 8 | 0 | 0 | 0 | 77 |
| Dundee nature reserve | June | 31 | 3 | 19 | 8 | 1 | 6 | 6 | 4 | 2 | 3940 |
| Dundee nature reserve | July | 45 | 4 | 35 | 5 | 1 | 4 | 4 | 4 | 0 | 5930 |
| Dundee nature reserve | August | 37 | 1 | 31 | 3 | 2 | 13 | 2 | 2 | 0 | 424 |
| Dundee nature reserve | September | 11 | 2 | 9 | 0 | 0 | 6 | 0 | 0 | 0 | 213 |
| Dundee urban | June | 20 | 3 | 8 | 7 | 2 | 0 | 4 | 4 | 1 | 214 |
| Dundee urban | July | 8 | 0 | 5 | 3 | 0 | 0 | 2 | 2 | 0 | 176 |
| Dundee urban | August | 19 | 0 | 14 | 5 | 0 | 8 | 3 | 2 | 0 | 206 |
| Dundee urban | September | 14 | 0 | 10 | 4 | 0 | 6 | 4 | 2 | 1 | 420 |
| Edinburgh farmland | June | 17 | 1 | 13 | 3 | 0 | 2 | 1 | 1 | 0 | 830 |
| Edinburgh farmland | July | 16 | 1 | 14 | 1 | 0 | 2 | 0 | 0 | 0 | 923 |
| Edinburgh farmland | August | 2 | 0 | 1 | 1 | 0 | 1 | 1 | 1 | 0 | 915 |
| Edinburgh farmland | September | 0 | 0 | 0 | 0 | 0 | 0 | 0 | 0 | 0 | 306 |
| Edinburgh nature reserve | June | 39 | 5 | 25 | 6 | 3 | 5 | 4 | 4 | 0 | 2813 |
| Edinburgh nature reserve | July | 6 | 0 | 4 | 2 | 0 | 1 | 1 | 1 | 0 | 1487 |
| Edinburgh nature reserve | August | 25 | 1 | 23 | 1 | 0 | 9 | 1 | 1 | 0 | 212 |
| Edinburgh nature reserve | September | 2 | 1 | 1 | 0 | 0 | 1 | 0 | 0 | 0 | 9 |
| Edinburgh urban | June | 7 | 0 | 2 | 5 | 0 | 0 | 5 | 5 | 0 | 135 |
| Edinburgh urban | July | 11 | 1 | 3 | 7 | 0 | 1 | 7 | 5 | 1 | 442 |
| Edinburgh urban | August | 10 | 0 | 6 | 4 | 0 | 4 | 4 | 3 | 1 | 41 |
| Edinburgh urban | September | 5 | 0 | 2 | 3 | 0 | 2 | 3 | 2 | 1 | 54 |
| Glasgow farmland | June | 28 | 3 | 21 | 1 | 3 | 7 | 1 | 1 | 0 | 372 |
| Glasgow farmland | July | 43 | 1 | 36 | 5 | 1 | 11 | 4 | 4 | 0 | 841 |
| Glasgow farmland | August | 24 | 0 | 22 | 2 | 0 | 8 | 1 | 1 | 0 | 290 |

| | | | | | | | | | | | |
|---|---|---|---|---|---|---|---|---|---|---|---|
| Glasgow farmland | September | 19 | 0 | 18 | 1 | 0 | 10 | 0 | 0 | 0 | 176 |
| Glasgow nature reserve | June | 32 | 10 | 17 | 4 | 1 | 6 | 2 | 2 | 0 | 515 |
| Glasgow nature reserve | July | 31 | 2 | 22 | 4 | 3 | 7 | 3 | 3 | 0 | 1505 |
| Glasgow nature reserve | August | 21 | 2 | 17 | 2 | 0 | 8 | 2 | 2 | 0 | 908 |
| Glasgow nature reserve | September | 30 | 3 | 24 | 3 | 0 | 11 | 1 | 1 | 0 | 423 |
| Glasgow urban | June | 3 | 0 | 2 | 1 | 0 | 1 | 1 | 1 | 0 | 280 |
| Glasgow urban | July | 3 | 0 | 3 | 0 | 0 | 0 | 0 | 0 | 0 | 196 |
| Glasgow urban | August | 8 | 0 | 6 | 2 | 0 | 1 | 0 | 0 | 0 | 175 |
| Glasgow urban | September | 4 | 0 | 4 | 0 | 0 | 4 | 0 | 0 | 0 | 73 |
| Hull farmland | June | 13 | 2 | 4 | 7 | 0 | 1 | 5 | 4 | 0 | 1703 |
| Hull farmland | July | 8 | 2 | 2 | 3 | 1 | 1 | 3 | 2 | 0 | 42 |
| Hull farmland | August | 11 | 0 | 9 | 2 | 0 | 8 | 1 | 1 | 0 | 375 |
| Hull farmland | September | 2 | 1 | 1 | 0 | 0 | 0 | 0 | 0 | 0 | 337 |
| Hull nature reserve | June | 24 | 0 | 16 | 8 | 0 | 10 | 6 | 6 | 0 | 229 |
| Hull nature reserve | July | 26 | 4 | 13 | 9 | 0 | 7 | 8 | 7 | 0 | 525 |
| Hull nature reserve | August | 44 | 3 | 31 | 9 | 1 | 14 | 7 | 6 | 0 | 1323 |
| Hull nature reserve | September | 22 | 1 | 20 | 1 | 0 | 7 | 1 | 1 | 0 | 351 |
| Hull urban | June | 9 | 1 | 5 | 3 | 0 | 0 | 3 | 3 | 0 | 76 |
| Hull urban | July | 6 | 0 | 2 | 4 | 0 | 1 | 4 | 4 | 0 | 286 |
| Hull urban | August | 15 | 0 | 12 | 3 | 0 | 8 | 3 | 1 | 1 | 1160 |
| Hull urban | September | 7 | 0 | 6 | 1 | 0 | 2 | 1 | 1 | 0 | 289 |
| Leeds farmland | June | 8 | 2 | 1 | 4 | 1 | 1 | 4 | 3 | 0 | 344 |
| Leeds farmland | July | 9 | 0 | 6 | 3 | 0 | 1 | 3 | 3 | 0 | 473 |
| Leeds farmland | August | 10 | 0 | 5 | 5 | 0 | 4 | 5 | 4 | 0 | 34 |
| Leeds farmland | September | 10 | 0 | 9 | 1 | 0 | 3 | 0 | 0 | 0 | 11 |
| Leeds nature reserve | June | 11 | 4 | 4 | 3 | 0 | 2 | 3 | 2 | 0 | 283 |

| | | | | | | | | | | | |
|---|---|---|---|---|---|---|---|---|---|---|---|
| Leeds nature reserve | July | 12 | 2 | 6 | 3 | 1 | 2 | 2 | 2 | 0 | 11 |
| Leeds nature reserve | August | 3 | 0 | 2 | 1 | 0 | 1 | 1 | 1 | 0 | 357 |
| Leeds nature reserve | September | 6 | 0 | 4 | 2 | 0 | 2 | 1 | 1 | 0 | 0 |
| Leeds urban | June | 15 | 0 | 4 | 11 | 0 | 2 | 10 | 8 | 1 | 3126 |
| Leeds urban | July | 5 | 0 | 2 | 3 | 0 | 1 | 2 | 1 | 0 | 1287 |
| Leeds urban | August | 13 | 0 | 8 | 5 | 0 | 8 | 5 | 3 | 1 | 369 |
| Leeds urban | September | 6 | 0 | 5 | 1 | 0 | 5 | 1 | 0 | 0 | 173 |
| London farmland | June | 11 | 2 | 2 | 7 | 0 | 2 | 5 | 4 | 0 | 1371 |
| London farmland | July | 12 | 4 | 3 | 3 | 2 | 2 | 2 | 1 | 0 | 190 |
| London farmland | August | 20 | 3 | 8 | 9 | 0 | 4 | 8 | 3 | 4 | 168 |
| London farmland | September | 5 | 0 | 3 | 2 | 0 | 2 | 1 | 1 | 0 | 9 |
| London nature reserve | June | 0 | 0 | 0 | 0 | 0 | 0 | 0 | 0 | 0 | 3 |
| London nature reserve | July | 2 | 0 | 1 | 1 | 0 | 1 | 1 | 1 | 0 | 18 |
| London nature reserve | August | 4 | 0 | 3 | 1 | 0 | 2 | 1 | 1 | 0 | 21 |
| London nature reserve | September | 4 | 0 | 3 | 1 | 0 | 3 | 1 | 1 | 0 | 41 |
| London urban | June | 15 | 2 | 6 | 6 | 1 | 2 | 6 | 5 | 0 | 123 |
| London urban | July | 33 | 4 | 14 | 14 | 1 | 6 | 11 | 4 | 6 | 148 |
| London urban | August | 17 | 4 | 5 | 8 | 0 | 4 | 8 | 2 | 5 | 38 |
| London urban | September | 11 | 0 | 4 | 7 | 0 | 1 | 6 | 3 | 2 | 58 |
| Reading farmland | June | 36 | 4 | 23 | 8 | 1 | 7 | 5 | 2 | 2 | 1295 |
| Reading farmland | July | 44 | 3 | 28 | 12 | 1 | 11 | 10 | 3 | 6 | 531 |
| Reading farmland | August | 1 | 0 | 1 | 0 | 0 | 0 | 0 | 0 | 0 | 6 |
| Reading farmland | September | 0 | 0 | 0 | 0 | 0 | 0 | 0 | 0 | 0 | 0 |
| Reading nature reserve | June | 2 | 1 | 0 | 1 | 0 | 0 | 1 | 1 | 0 | 519 |
| Reading nature reserve | July | 8 | 0 | 6 | 2 | 0 | 6 | 2 | 1 | 1 | 189 |

| | | | | | | | | | | | |
|---|---|---|---|---|---|---|---|---|---|---|---|
| Reading nature reserve | August | 6 | 0 | 3 | 3 | 0 | 1 | 3 | 2 | 0 | 3501 |
| Reading nature reserve | September | 6 | 0 | 3 | 3 | 0 | 3 | 3 | 2 | 0 | 2081 |
| Reading urban | June | 12 | 2 | 6 | 4 | 0 | 2 | 4 | 4 | 0 | 271 |
| Reading urban | July | 26 | 1 | 12 | 13 | 0 | 7 | 12 | 4 | 7 | 149 |
| Reading urban | August | 6 | 0 | 3 | 3 | 0 | 1 | 3 | 2 | 0 | 43 |
| Reading urban | September | 7 | 0 | 4 | 3 | 0 | 2 | 3 | 1 | 1 | 19 |
| Sheffield farmland | June | 9 | 2 | 5 | 2 | 0 | 0 | 2 | 1 | 1 | 135 |
| Sheffield farmland | July | 8 | 1 | 3 | 4 | 0 | 1 | 2 | 2 | 0 | 109 |
| Sheffield farmland | August | 5 | 0 | 3 | 2 | 0 | 3 | 2 | 1 | 0 | 38 |
| Sheffield farmland | September | 10 | 0 | 8 | 2 | 0 | 5 | 2 | 2 | 0 | 6 |
| Sheffield nature reserve | June | 0 | 0 | 0 | 0 | 0 | 0 | 0 | 0 | 0 | 0 |
| Sheffield nature reserve | July | 0 | 0 | 0 | 0 | 0 | 0 | 0 | 0 | 0 | 22 |
| Sheffield nature reserve | August | 15 | 0 | 10 | 5 | 0 | 9 | 3 | 2 | 0 | 3297 |
| Sheffield nature reserve | September | 1 | 0 | 0 | 1 | 0 | 0 | 1 | 1 | 0 | 16787 |
| Sheffield urban | June | 9 | 0 | 2 | 7 | 0 | 2 | 7 | 5 | 1 | 969 |
| Sheffield urban | July | 7 | 0 | 2 | 5 | 0 | 1 | 5 | 2 | 2 | 229 |
| Sheffield urban | August | 8 | 2 | 3 | 2 | 1 | 3 | 2 | 2 | 0 | 249 |
| Sheffield urban | September | 5 | 0 | 4 | 1 | 0 | 4 | 1 | 1 | 0 | 69 |
| Southampton farmland | June | 1 | 0 | 1 | 0 | 0 | 0 | 0 | 0 | 0 | 229 |
| Southampton farmland | July | 29 | 2 | 20 | 7 | 0 | 11 | 7 | 2 | 4 | 646 |
| Southampton farmland | August | 24 | 1 | 16 | 6 | 1 | 2 | 5 | 2 | 2 | 268 |
| Southampton farmland | September | 26 | 0 | 21 | 5 | 0 | 9 | 4 | 2 | 1 | 135 |
| Southampton nature reserve | June | 1 | 0 | 1 | 0 | 0 | 1 | 0 | 0 | 0 | 31 |

| | | | | | | | | | | | |
|---|---|---|---|---|---|---|---|---|---|---|---|
| Southampton nature reserve | July | 4 | 0 | 2 | 2 | 0 | 1 | 2 | 1 | 1 | 11 |
| Southampton nature reserve | August | 1 | 0 | 0 | 1 | 0 | 0 | 1 | 0 | 0 | 59 |
| Southampton nature reserve | September | 5 | 0 | 3 | 2 | 0 | 2 | 2 | 1 | 0 | 9 |
| Southampton urban | June | 6 | 0 | 1 | 5 | 0 | 1 | 5 | 4 | 0 | 174 |
| Southampton urban | July | 10 | 1 | 2 | 7 | 0 | 2 | 6 | 3 | 2 | 161 |
| Southampton urban | August | 6 | 0 | 3 | 3 | 0 | 2 | 3 | 2 | 0 | 61 |
| Southampton urban | September | 5 | 0 | 2 | 3 | 0 | 1 | 3 | 2 | 1 | 30 |
| Swindon farmland | June | 1 | 0 | 0 | 1 | 0 | 0 | 0 | 0 | 0 | 65 |
| Swindon farmland | July | 0 | 0 | 0 | 0 | 0 | 0 | 0 | 0 | 0 | 29 |
| Swindon farmland | August | 7 | 1 | 5 | 1 | 0 | 3 | 1 | 0 | 0 | 24 |
| Swindon farmland | September | 8 | 0 | 7 | 1 | 0 | 3 | 0 | 0 | 0 | 8 |
| Swindon nature reserve | June | 9 | 2 | 6 | 1 | 0 | 0 | 0 | 0 | 0 | 171 |
| Swindon nature reserve | July | 18 | 1 | 9 | 3 | 5 | 3 | 3 | 2 | 1 | 138 |
| Swindon nature reserve | August | 33 | 2 | 18 | 9 | 4 | 7 | 7 | 4 | 2 | 47 |
| Swindon nature reserve | September | 37 | 3 | 23 | 9 | 2 | 4 | 4 | 3 | 0 | 72 |
| Swindon urban | June | 7 | 1 | 2 | 3 | 1 | 1 | 3 | 2 | 1 | 72 |
| Swindon urban | July | 13 | 1 | 7 | 5 | 0 | 3 | 5 | 4 | 0 | 205 |
| Swindon urban | August | 15 | 0 | 8 | 6 | 1 | 4 | 6 | 5 | 0 | 95 |
| Swindon urban | September | 9 | 0 | 6 | 3 | 0 | 4 | 3 | 3 | 0 | 39 |

**Table 5 Inverse Simpsons and Fisher's Alpha indices calculated for visitor communities per site; floral unit abundance recorded across whole sampling season per site; proportion of woodland habitat at site; Sørenson similarity index, proportional similarity and Horn Morisita index calculated for the visitor community at each site.**

| Site | Inverse Simpson's | Fisher's Alpha | Floral Unit Abundance | Proportion woodland habitat | Sørenson Index | Proportional similarity | Horn Morisita Index | Visitor generality | Plant generality | Network specialisation (H2') |
|---|---|---|---|---|---|---|---|---|---|---|
| Bristol farmland | 12.583 | 24.231 | 715 | 0.095 | 0.350 | 0.290 | 0.622 | 2.399 | 8.553 | 0.509 |
| Bristol nature reserve | 25.786 | 25.985 | 307 | 0.271 | 0.283 | 0.197 | 0.767 | 2.257 | 10.244 | 0.488 |
| Bristol urban | 6.063 | 15.747 | 1314 | 0.056 | 0.381 | 0.346 | 0.608 | 4.464 | 2.730 | 0.482 |
| Cardiff farmland | 5.935 | 26.381 | 2021 | 0.075 | 0.311 | 0.233 | 0.722 | 3.175 | 8.512 | 0.497 |
| Cardiff nature reserve | 8.794 | 19.315 | 1600 | 0.000 | 0.282 | 0.211 | 0.772 | 3.214 | 6.541 | 0.466 |
| Cardiff urban | 7.767 | 14.324 | 473 | 0.169 | 0.340 | 0.178 | 0.874 | 2.206 | 4.471 | 0.574 |
| Dundee farmland | 9.469 | 29.329 | 2533 | 0.046 | 0.279 | 0.235 | 0.628 | 1.829 | 14.811 | 0.589 |
| Dundee nature reserve | 19.859 | 31.767 | 10507 | 0.238 | 0.215 | 0.170 | 0.743 | 2.406 | 10.656 | 0.571 |
| Dundee urban | 6.012 | 18.039 | 1016 | 0.028 | 0.389 | 0.351 | 0.469 | 3.626 | 3.759 | 0.485 |
| Edinburgh farmland | 7.668 | 11.983 | 2974 | 0.149 | 0.230 | 0.233 | 0.646 | 3.284 | 4.257 | 0.477 |
| Edinburgh nature reserve | 6.632 | 22.969 | 4521 | 0.391 | 0.234 | 0.247 | 0.591 | 3.307 | 4.005 | 0.544 |
| Edinburgh urban | 5.086 | 10.076 | 672 | 0.077 | 0.371 | 0.400 | 0.421 | 4.278 | 2.425 | 0.552 |
| Glasgow farmland | 7.759 | 27.485 | 1679 | 0.280 | 0.238 | 0.142 | 0.901 | 1.748 | 11.162 | 0.667 |
| Glasgow nature reserve | 18.372 | 32.094 | 3351 | 0.629 | 0.250 | 0.159 | 0.854 | 2.836 | 6.537 | 0.580 |
| Glasgow urban | 11.255 | 29.468 | 724 | 0.006 | 0.199 | 0.218 | 0.571 | 1.228 | 4.326 | 0.641 |
| Hull farmland | 10.489 | 14.863 | 2457 | 0.139 | 0.320 | 0.275 | 0.580 | 1.891 | 5.197 | 0.591 |
| Hull nature reserve | 9.270 | 20.321 | 2428 | 0.033 | 0.273 | 0.308 | 0.542 | 1.829 | 14.811 | 0.589 |
| Hull urban | 5.736 | 8.468 | 1811 | 0.000 | 0.411 | 0.346 | 0.601 | 3.687 | 2.563 | 0.598 |
| Leeds farmland | 9.191 | 8.981 | 862 | 0.315 | 0.269 | 0.283 | 0.567 | 1.093 | 5.529 | 0.908 |
| Leeds nature reserve | 12.252 | 18.074 | 651 | 0.960 | 0.262 | 0.237 | 0.653 | 1.454 | 7.058 | 0.636 |
| Leeds urban | 9.667 | 10.081 | 4955 | 0.121 | 0.431 | 0.451 | 0.386 | 5.200 | 3.741 | 0.419 |
| London farmland | 7.646 | 12.255 | 1738 | 0.112 | 0.266 | 0.248 | 0.650 | 2.506 | 5.577 | 0.481 |
| London nature reserve | 1.998 | 2.931 | 83 | 0.878 | 0.189 | 0.229 | 0.620 | 2.251 | 1.973 | 0.428 |
| London urban | 17.252 | 24.321 | 367 | 0.081 | 0.324 | 0.349 | 0.547 | 4.341 | 6.455 | 0.354 |

| | | | | | | | | | | |
|---|---|---|---|---|---|---|---|---|---|---|
| Reading farmland | 19.834 | 28.672 | 1832 | 0.162 | 0.315 | 0.283 | 0.556 | 1.924 | 12.884 | 0.571 |
| Reading nature reserve | 7.327 | 8.929 | 6290 | 0.841 | 0.264 | 0.313 | 0.534 | 1.663 | 6.911 | 0.503 |
| Reading urban | 12.578 | 22.239 | 482 | 0.119 | 0.425 | 0.441 | 0.402 | 3.837 | 3.488 | 0.288 |
| Sheffield farmland | 11.664 | 19.714 | 288 | 0.179 | 0.253 | 0.274 | 0.588 | 1.903 | 5.244 | 0.4815 |
| Sheffield nature reserve[a] | 4.582 | 3.628 | 20106 | 0.000 | 0.253 | 0.305 | 0.543 | 1.000 | 6.433 | - |
| Sheffield urban | 3.454 | 7.687 | 1516 | 0.006 | 0.375 | 0.395 | 0.568 | 4.934 | 3.027 | 0.382 |
| Southampton farmland | 13.413 | 19.92 | 1278 | 0.005 | 0.299 | 0.291 | 0.591 | 3.159 | 8.020 | 0.457 |
| Southampton nature reserve | 6.125 | 7.757 | 110 | 0.819 | 0.173 | 0.267 | 0.564 | 1.808 | 2.282 | 0.286 |
| Southampton urban | 4.963 | 5.225 | 426 | 0.007 | 0.400 | 0.391 | 0.464 | 6.572 | 2.161 | 0.435 |
| Swindon farmland | 12.000 | 17.115 | 126 | 0.111 | 0.132 | 0.180 | 0.679 | 1.222 | 4.579 | 0.728 |
| Swindon nature reserve | 8.501 | 21.011 | 428 | 0.022 | 0.268 | 0.163 | 0.793 | 2.456 | 10.795 | 0.458 |
| Swindon urban | 8.656 | 12.739 | 411 | 0.094 | 0.392 | 0.411 | 0.466 | 3.113 | 4.268 | 0.433 |

[a] Note it was not possible to calculate H2’ for the Sheffield nature reserve site as the network was too small (one plant species only), thus analyses for comparisons of H2’ across landscape types excludes the triplet of sites for Sheffield.

**Table 6 Visitor generality, plant generality, network specialisation (H2'), visitor species-level specialisation (d') and plant species-level specialisation (d') calculated for the flower-visitor network at each site.**

| Site | Visitor generality | Plant generality | Network specialisation (H2') | Visitor specialisation (d') | Visitor specialisation (d') |
|---|---|---|---|---|---|
| Bristol farmland | 2.399 | 8.553 | 0.509 | 0.306 | 0.614 |
| Bristol nature reserve | 2.257 | 10.244 | 0.488 | 0.325 | 0.540 |
| Bristol urban | 4.464 | 2.730 | 0.482 | 0.667 | 0.597 |
| Cardiff farmland | 3.175 | 8.512 | 0.497 | 0.256 | 0.564 |
| Cardiff nature reserve | 3.214 | 6.541 | 0.466 | 0.288 | 0.486 |
| Cardiff urban | 2.206 | 4.471 | 0.574 | 0.478 | 0.749 |
| Dundee farmland | 1.829 | 14.811 | 0.589 | 0.186 | 0.463 |
| Dundee nature reserve | 2.406 | 10.656 | 0.571 | 0.306 | 0.560 |
| Dundee urban | 3.626 | 3.759 | 0.485 | 0.443 | 0.509 |
| Edinburgh farmland | 3.284 | 4.257 | 0.477 | 0.318 | 0.440 |
| Edinburgh nature reserve | 3.307 | 4.005 | 0.544 | 0.452 | 0.551 |
| Edinburgh urban | 4.278 | 2.425 | 0.552 | 0.498 | 0.415 |
| Glasgow farmland | 1.748 | 11.162 | 0.667 | 0.284 | 0.654 |
| Glasgow nature reserve | 2.836 | 6.537 | 0.580 | 0.440 | 0.609 |
| Glasgow urban | 1.228 | 4.326 | 0.641 | 0.358 | 0.797 |
| Hull farmland | 1.891 | 5.197 | 0.591 | 0.345 | 0.687 |
| Hull nature reserve | 1.829 | 14.811 | 0.589 | 0.186 | 0.463 |
| Hull urban | 3.687 | 2.563 | 0.598 | 0.596 | 0.593 |
| Leeds farmland | 1.093 | 5.529 | 0.908 | 0.391 | 0.874 |
| Leeds nature reserve | 1.454 | 7.058 | 0.636 | 0.312 | 0.700 |
| Leeds urban | 5.200 | 3.741 | 0.419 | 0.471 | 0.393 |
| London farmland | 2.506 | 5.577 | 0.481 | 0.327 | 0.483 |
| London nature reserve | 2.251 | 1.973 | 0.428 | 0.468 | 0.370 |
| London urban | 4.341 | 6.455 | 0.354 | 0.415 | 0.446 |
| Reading farmland | 1.924 | 12.884 | 0.571 | 0.242 | 0.593 |

| | | | | | |
|---|---|---|---|---|---|
| Reading nature reserve | 1.663 | 6.911 | 0.503 | 0.192 | 0.550 |
| Reading urban | 3.837 | 3.488 | 0.288 | 0.457 | 0.517 |
| Sheffield farmland | 1.903 | 5.244 | 0.482 | 0.359 | 0.632 |
| Sheffield nature reserve[a] | 1.000 | 6.433 | - | - | - |
| Sheffield urban | 4.934 | 3.027 | 0.382 | 0.316 | 0.337 |
| Southampton farmland | 3.159 | 8.020 | 0.457 | 0.287 | 0.469 |
| Southampton nature reserve | 1.808 | 2.282 | 0.286 | 0.446 | 0.514 |
| Southampton urban | 6.572 | 2.161 | 0.435 | 0.604 | 0.389 |
| Swindon farmland | 1.222 | 4.579 | 0.728 | 0.314 | 0.713 |
| Swindon nature reserve | 2.456 | 10.795 | 0.458 | 0.217 | 0.566 |
| Swindon urban | 3.113 | 4.268 | 0.433 | 0.417 | 0.461 |

[a] Note it was not possible to calculate H2’ or d’ for the Sheffield nature reserve site as the network was too small (one plant species only), thus analyses for comparisons of H2’ and d’ across landscape types excludes the triplet of sites for Sheffield.

**Table 7 Number of local singleton (recorded once at triplet of urban, farmland and nature reserve sites for each city), locally rare (recorded 2 to 10 times), locally less common (recorded 11 to 50 times) and locally common (recorded >50 times) visitor taxa for urban, farmland and nature reserve sites. Values are also expressed as a proportion of the total number of taxa found for the 12 sites of that landscape type.**

| | Number | | | | Proportion | | | |
|---|---|---|---|---|---|---|---|---|
| Site | Singleton | Rare | Less common | Common | Singleton | Rare | Less common | Common |
| Bristol farmland | 21 | 28 | 12 | 2 | 0.33 | 0.44 | 0.19 | 0.03 |
| Bristol nature reserve | 13 | 23 | 12 | 2 | 0.26 | 0.46 | 0.24 | 0.04 |
| Bristol urban | 12 | 19 | 10 | 1 | 0.29 | 0.45 | 0.24 | 0.02 |
| Cardiff farmland | 23 | 38 | 17 | 2 | 0.29 | 0.48 | 0.21 | 0.03 |
| Cardiff nature reserve | 16 | 20 | 15 | 2 | 0.30 | 0.38 | 0.28 | 0.04 |
| Cardiff urban | 10 | 12 | 14 | 2 | 0.26 | 0.32 | 0.37 | 0.05 |
| Dundee farmland | 21 | 36 | 17 | 0 | 0.28 | 0.48 | 0.23 | 0.00 |
| Dundee nature reserve | 27 | 37 | 16 | 0 | 0.33 | 0.46 | 0.20 | 0.00 |
| Dundee urban | 11 | 22 | 12 | 0 | 0.24 | 0.48 | 0.26 | 0.00 |
| Edinburgh farmland | 11 | 12 | 4 | 0 | 0.41 | 0.44 | 0.15 | 0.00 |
| Edinburgh nature reserve | 27 | 26 | 6 | 1 | 0.45 | 0.43 | 0.10 | 0.02 |
| Edinburgh urban | 7 | 12 | 4 | 0 | 0.30 | 0.52 | 0.17 | 0.00 |
| Glasgow farmland | 3 | 59 | 13 | 0 | 0.04 | 0.79 | 0.17 | 0.00 |
| Glasgow nature reserve | 8 | 57 | 16 | 0 | 0.10 | 0.70 | 0.20 | 0.00 |
| Glasgow urban | 0 | 11 | 6 | 0 | 0.00 | 0.65 | 0.35 | 0.00 |
| Hull farmland | 9 | 10 | 6 | 4 | 0.31 | 0.34 | 0.21 | 0.14 |
| Hull nature reserve | 28 | 30 | 12 | 4 | 0.38 | 0.41 | 0.16 | 0.05 |
| Hull urban | 6 | 7 | 8 | 4 | 0.24 | 0.28 | 0.32 | 0.16 |
| Leeds farmland | 8 | 8 | 7 | 0 | 0.35 | 0.35 | 0.30 | 0.00 |
| Leeds nature reserve | 9 | 10 | 5 | 0 | 0.38 | 0.42 | 0.21 | 0.00 |
| Leeds urban | 8 | 12 | 7 | 0 | 0.30 | 0.44 | 0.26 | 0.00 |
| London farmland | 9 | 18 | 7 | 1 | 0.26 | 0.51 | 0.20 | 0.03 |
| London nature reserve | 2 | 3 | 2 | 0 | 0.29 | 0.43 | 0.29 | 0.00 |
| London urban | 20 | 23 | 7 | 1 | 0.39 | 0.45 | 0.14 | 0.02 |

| | | | | | | | | |
|---|---|---|---|---|---|---|---|---|
| Reading farmland | 26 | 29 | 5 | 0 | 0.43 | 0.48 | 0.08 | 0.00 |
| Reading nature reserve | 3 | 10 | 3 | 0 | 0.19 | 0.63 | 0.19 | 0.00 |
| Reading urban | 10 | 21 | 4 | 0 | 0.29 | 0.60 | 0.11 | 0.00 |
| Sheffield farmland | 14 | 6 | 3 | 2 | 0.56 | 0.24 | 0.12 | 0.08 |
| Sheffield nature reserve | 2 | 7 | 3 | 2 | 0.14 | 0.50 | 0.21 | 0.14 |
| Sheffield urban | 8 | 6 | 3 | 2 | 0.42 | 0.32 | 0.16 | 0.11 |
| Southampton farmland | 20 | 24 | 7 | 0 | 0.39 | 0.47 | 0.14 | 0.00 |
| Southampton nature reserve | 2 | 2 | 3 | 0 | 0.29 | 0.29 | 0.43 | 0.00 |
| Southampton urban | 3 | 8 | 3 | 0 | 0.21 | 0.57 | 0.21 | 0.00 |
| Swindon farmland | 5 | 8 | 2 | 0 | 0.33 | 0.53 | 0.13 | 0.00 |
| Swindon nature reserve | 21 | 33 | 10 | 1 | 0.32 | 0.51 | 0.15 | 0.02 |
| Swindon urban | 7 | 14 | 6 | 0 | 0.26 | 0.52 | 0.22 | 0.00 |

**Table 8 Overall, native and non-native flowering plant richness per site; overall, native and non-native floral unit abundance per site**

| | Flowering plant richness | | | Floral unit abundance | | |
|---|---|---|---|---|---|---|
| Site | All | Non-native | Native | All | Non-native | Native |
| Bristol farmland | 23 | 0 | 23 | 715 | 715 | 0 |
| Bristol nature reserve | 23 | 0 | 23 | 307 | 307 | 0 |
| Bristol urban | 55 | 34 | 21 | 1314 | 384 | 930 |
| Cardiff farmland | 28 | 0 | 28 | 2021 | 2021 | 0 |
| Cardiff nature reserve | 32 | 0 | 32 | 1600 | 1600 | 0 |
| Cardiff urban | 32 | 16 | 16 | 473 | 94 | 379 |
| Dundee farmland | 23 | 4 | 19 | 2533 | 1287 | 1246 |
| Dundee nature reserve | 32 | 1 | 31 | 10507 | 10507 | 0 |
| Dundee urban | 54 | 27 | 27 | 1016 | 365 | 651 |
| Edinburgh farmland | 31 | 2 | 29 | 2974 | 2967 | 7 |
| Edinburgh nature reserve | 40 | 0 | 40 | 4521 | 4521 | 0 |
| Edinburgh urban | 42 | 20 | 22 | 672 | 426 | 246 |
| Glasgow farmland | 30 | 1 | 29 | 1679 | 1610 | 69 |
| Glasgow nature reserve | 52 | 2 | 50 | 3351 | 3109 | 242 |
| Glasgow urban | 24 | 4 | 20 | 724 | 636 | 88 |
| Hull farmland | 26 | 2 | 24 | 2457 | 873 | 1584 |
| Hull nature reserve | 48 | 1 | 47 | 2428 | 1992 | 436 |
| Hull urban | 40 | 13 | 27 | 1811 | 1736 | 75 |
| Leeds farmland | 11 | 1 | 10 | 862 | 862 | 0.00 |
| Leeds nature reserve | 15 | 1 | 14 | 651 | 648 | 3 |
| Leeds urban | 48 | 13 | 35 | 4955 | 3528 | 1427 |
| London farmland | 16 | 3 | 13 | 1738 | 223 | 1515 |
| London nature reserve | 10 | 0 | 10 | 83 | 83 | 0.00 |
| London urban | 46 | 22 | 24 | 367 | 316 | 51 |
| Reading farmland | 17 | 1 | 16 | 1832 | 1830 | 2 |
| Reading nature reserve | 5 | 0 | 5 | 6290 | 6290 | 0.00 |

| | | | | | | |
|---|---|---|---|---|---|---|
| Reading urban | 48 | 17 | 31 | 482 | 362 | 120 |
| Sheffield farmland | 15 | 1 | 14 | 288 | 272 | 16 |
| Sheffield nature reserve | 2 | 0 | 2 | 20106 | 20106 | 0 |
| Sheffield urban | 36 | 12 | 24 | 1516 | 1326 | 190 |
| Southampton farmland | 33 | 2 | 31 | 1278 | 1274 | 4 |
| Southampton nature reserve | 8 | 0 | 8 | 110 | 110 | 0 |
| Southampton urban | 44 | 28 | 16 | 426 | 259 | 167 |
| Swindon farmland | 11 | 1 | 10 | 126 | 111 | 15 |
| Swindon nature reserve | 17 | 0 | 17 | 428 | 428 | 0 |
| Swindon urban | 26 | 14 | 12 | 411 | 256 | 155 |
